# A systematic review and meta-analysis of the prevalence of tick-borne SFGR in China from 2000 to 2022

**DOI:** 10.1371/journal.pntd.0012550

**Published:** 2024-10-09

**Authors:** Yanchao Hu, Ting Yin, Wei Ma, Jiangwei Qiu, Jiaxing Zhang, Qingan Wang

**Affiliations:** 1 Department of Infectious Disease, General Hospital of Ningxia Medical University, Yinchuan, China; 2 Department of Epidemiology and Health Statistics, School of Public Health, Ningxia Medical University, Yinchuan, China; 3 Key Laboratory of Environmental Factors and Chronic Disease Control, Ningxia Medical University, Yinchuan, China; 4 Department of Dermatology, The Second People’s Hospital of Yinchuan, Yinchuanf, China; Egerton University - Njoro Campus: Egerton University, KENYA

## Abstract

**Background:**

Ticks carry and transmit a wide range of pathogens (bacteria, viruses, and protozoa) that pose significant threats to human and animal health worldwide. Only few meta-analyses have been conducted on the distribution of ticks and tick-borne spotted fever group rickettsia (SFGR). Therefore, this study aims to examine the tick species and SFGR positivity in China in order to provide support for further research and improvements in the prevention and control of tick-borne diseases.

**Methodology:**

This systematic review was performed in accordance with the Preferred Reporting Items for Systematic Reviews and Meta-Analyses (PRISMA) guidelines. Relevant Chinese and English studies were retrieved from PubMed, Embase, Cochrane Library, Web of Science, China National Knowledge Infrastructure (CNKI), VIP database, Chinese Biomedical literature database (CBM) and Wanfang database from inception to January 9, 2023. Pooled SFGR positive rate was meta-analyzed using a random effects model and heterogeneity was assessed by the I2 index. Publication bias was also evaluated by funnel plot and Egger’s test. The meta-analysis was performed on R studio 4.0.4.

**Principal findings:**

Meta-analysis of 57 studies published between 2000 and 2022 involving 39,380 ticks revealed a pooled SFGR positive rate of 21.4% (Q = 6423.74, I^2^ = 99%, Q-*p*<0.001, 95% CI: 15.0–29.6). Most studies of tick-borne SFGR infection rate were conducted in forest areas and developed animal husbandry areas in the northern region. There were slightly more tick species in the southern region, but the differences in tick species (Feeding tick 31.5%, 95%CI: 15.7–53.2, Questing tick 11.5%, 95%CI: 4.4–26.7, Q = 3.29, Q-*p* = 0.19) between areas (Northern area 20.4%, 95%CI: 14.1–28.7, Southern area 25.5%, 95%CI: 15.0–29.6, Q = 0.21, Q-*p* = 0.64) were not statistically significant. The most common tick species were *Dermacentor silvarum* (13%), *Ixodes persulcatus* (11%) and *Haemaphysalis Iongicornis* (10%), and the most prevalent SFGR species were *Rickettsia raoultii* (20%), *Rickettsia heilongjiangiensis* (11%), and some uncultured species (18%).

**Conclusions:**

This study examined the distribution of tick-borne SFGR in China. Our findings revealed that the main tick species were *D*. *silvarum*, *I*. *persulcatus* and *H*. *iongicornis*, and the common SFGR species were *R*. *raoultii*, *R*. *heilongjiangiensis*, and some uncultured species. Further studies are warranted to identify the potential vectors of SFGR and to better understand the epidemiology and pathogenesis of tick-borne diseases in China.

## Author summary

Tick-borne rickettsial diseases caused by spotted fever group rickettsia (SFGR) are natural zoonotic diseases that seriously threaten human health worldwide, which mainly transmitted to humans and animals by tick bites.

This is the first study systematically examined the distribution of tick-borne spotted fever group rickettsia (SFGR) in China. To analyze the positive rates of tick-borne SFGR in China, we conducted a meta-analysis of 57 studies reporting 39,380 ticks that were published in Chinese and English between 2000 and 2022. Setting the Qinling Mountains-Huaihe River Line as the boundary between northern and southern China, the main tick species in China are *D*. *silvarum*, *I*. *persulcatus* and *H*. *Iongicornis*, and the common SFGR species they carry are *R*. *raoultii*, *R*. *heilongjiangiensis*, and some unclassified species. The findings highlight awareness of future tick-borne disease prevention and control strategies, such as vaccination programs and public awareness campaigns.

## 1. Introduction

Spotted fever group of rickettsia (SFGR) are a group of tick-borne obligate intracellular bacteria that cause a variety of natural zoonotic diseases with fever as the main symptom [1], such as Mediterranean Spotted Fever in parts of Europe and Africa, Rocky Mountain Spotted Fever in the United States, and Heilongjiang Spotted Fever and Inner Mongolia Spotted Fever in China [2–4]. A total of 33 new tick-borne pathogens have been reported in China since 1982, including 8 new SFGR species, but the pathogenesis of some rickettsia species is still unclear [5]. Therefore, tick-borne rickettsioses pose major challenges to healthcare worldwide. Some studies have confirmed that certain rickettsia species can survive in different developmental stages and are transmitted to the host through the salivary glands via bites [6,7]. SFGR was first isolated in 1962 from ticks and animals in Hulin, Heilongjiang Province, and was subsequently studied in most provinces of China [8,9].

The increase in the prevalence and transmission of tick-borne diseases is a serious public health concern. In the last 40 years, at least 22 diseases caused by tick-borne pathogens have been reported worldwide, and over 28 tick species are known to cause various human diseases, such as Lyme disease and SFGR [10,11]. Persistent recurrent infections caused by tick-borne pathogens and sequelae from long-term infections further deteriorate the quality of human health and can even lead to death through misdiagnosis and delayed treatment [12]. In addition, the global economic burden on livestock has also increased as a consequence of tick-borne pathogens [13]. SFGR is an obligatory intracellular parasite of Rickettsia genus [14] and an important pathogen of tick-borne and zoonotic diseases. Novel rickettsia species whose pathogenicity has not yet been determined are continued to be found in or isolated from ticks around the world [15,16]. The dominant tick species varies among different habitats in the northern and southern regions of China, and this in turn results in the carrying of diverse pathogens [17,18]. Suitable climate and environment provide favorable conditions for the survival of ticks and host animals, and further promote the spread of SFGR [19]. China is vast in area and varied in terrain, spanning nearly three time zones from east to west, including tropical, warm temperate, temperate, and cold temperate zones in the north and south, with a diverse range of tick species. Here, we conducted a meta-analysis of 57 Chinese and English articles involving 39,380 ticks that were published between 2000 and 2022. The present systematic review and meta-analysis is aimed to examine the distribution and molecular epidemiology of tick-borne Rickettsia. This is the first report to date that analyzed the positive rate of tick-borne SFGR in different provinces of China, offering new insights into the prevention and control of spotted fever.

## 2. Methods

This systematic review and meta-analysis were structured in accordance with the recommendations for Preferred Reporting Items for Systematic Reviews and Meta-Analyses (PRISMA) and has been registered in the International Prospective Register of Systematic Reviews (PROSPERO; identifier CRD42023448420, https://www.crd.york.ac.uk/PROSPERO).

### 2.1 Search strategy

Relevant studies published between January 1, 2000 and December 31, 2022 were independently searched by two researchers from eight databases, including four English databases (PubMed, Embase, Cochrane Library and Web of Science) and four Chinese databases (China National Knowledge Infrastructure (CNKI), VIP, Chinese Biomedical literature and Wanfang). The search keywords were China, Tick, Spotted Fever Group of Rickettsia, prevalence, incidence and epidemiology. The detailed search strategy is shown in S1 Table.

The systematic search was accomplished following the combination of questing text and wordlist terms in diverse distinctions: (‘Tick’ OR, ‘Ixodidae’)AND(‘Spotted Fever Group Rickettsiosis’ OR ‘spotted fever rickettsiae disease’). Thereafter, titles and abstract were screened and potential journal articles were reviewed and downloaded. The references of potential articles were also reviewed to identify additional relevant articles.

#### 2.1.1 Inclusion criteria

Studies were considered eligible for the meta-analysis if they: (1) were original published studies in China investigating the occurrence of SFGR in ticks; (2) utilized molecular based methods to screen for tick-borne SFGR; (3) reported the source and species of ticks and identified the SFGR species; (4) clearly quantified the number of SFGR-positive ticks; (5) were published between January 1, 2000 and December 31, 2022; and (6) published in both English and Chinese languages.

#### 2.1.2 Exclusion criteria

Studies were excluded if they meet the following criteria: (1) meta-analysis, reviews and letters; (2) not published in Chinese or English; (3) non-epidemiological studies or population/animal serological studies.

#### 2.1.3 Selection criteria

All potential studies were first imported into EndNote 20 to remove duplications. Titles and abstracts were screened independently by two researchers (TY and YC H) according to the eligibility criteria, and the full-texts of eligible studies were further assessed. Disagreements between the evaluators were resolved by consensus or discussion with a third researcher (WM).

### 2.2 Data extraction

Data extracted included article title, name of first author, name of corresponding author, year of publication, journal of publication, research year, research region, total sample size, tick species, number of detected ticks, SFGR positivity, detection method, and specific SFGR species. The extracted data are summarized in S1 and S2 Tables.

### 2.3 Quality assessment

Quality of the included studies was assessed independently by two researchers (TY and YC H) using the Risk of Bias Tool by Hoy et al. The risk of bias is assessed across 10 domains, each given a score of 0 or 1 to indicate the absence or presence of bias. A total score of 0–3 is regarded as low risk of bias, 4–6 as moderate risk of bias, and 7–10 as high risk of bias [20].

### 2.4 Statistical analysis

All included studies were descriptively summarized in this meta-analysis, and SFGR infection rates were logit-transformed, which helps to normalize data distribution and ensure the validity of subsequent analyses. After the transformation, infection rates and their corresponding 95% confidence intervals (CIs) were calculated. Heterogeneity among studies was assessed using the *I*^2^ index. When *I*^2^ < 50% (low heterogeneity), a fixed effect model was used for meta-analysis; otherwise, a random effects model was utilized. In addition, subgroup and meta-regression analyses were performed to explore potential sources of heterogeneity and assess the impact of various factors on SFGR infection rates.

Publication bias was evaluated using funnel plot, and Egger’s test was performed to assess funnel plot asymmetry. Sensitivity analyses were conducted to examine the robustness of the pooled results. Meta-analysis was conducted using the ‘meta’ and ‘metafor’ packages in R studio 4.0.4. Data analysis and georeferenced of SFGR infection rates on an epidemic map of China were completed in SPSS 24.0 and ArcGIS 10.7, respectively. A *P*<0.05 was considered statistically significant.

## 3. Results

### 3.1 Literature selection

**Fig 1:**
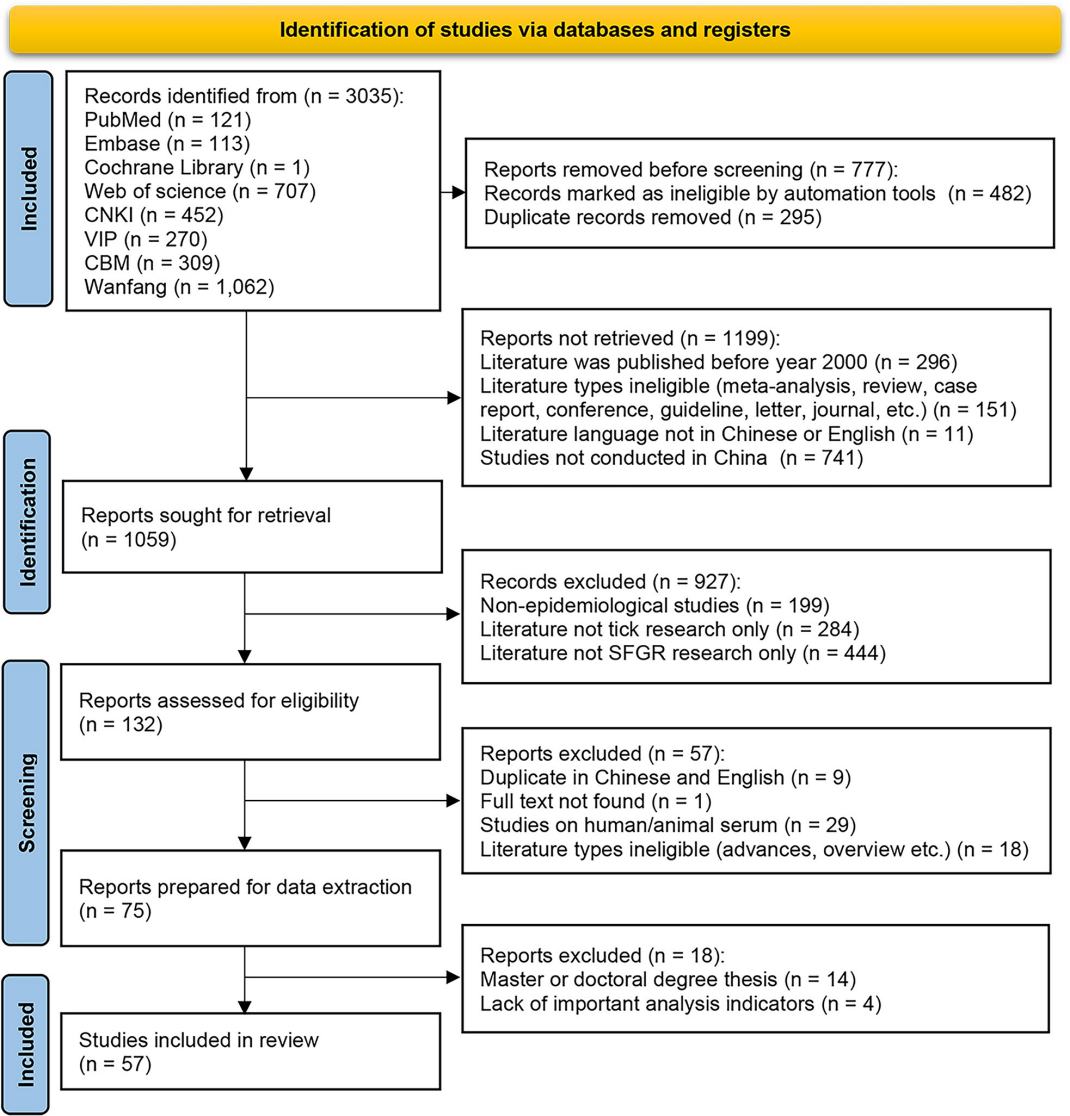
Flow diagram of search strategy.

Our initial search yielded 3,035 articles that investigated the prevalence of tick-borne SFGR in China. We imported the studies into Endnote and removed a total of 1,976 articles due to duplication and meeting the exclusion criteria. The full texts of the remaining studies were screened, and 1,002 articles were further removed due to being non-epidemiological studies(n = 199), human/animal serological studies(n = 39), master or doctoral thesis(n = 36), and studies that investigated not only tick or SFGR(n = 728). A final total of 57 eligible studies describing 39,380 tick species and detection and classification of SFGR were included in our meta-analysis (Fig 1).

### 3.2 General characteristics and quality of included studies

The 57 included studies covered most areas of northern and southern China. The types of data reported in these studies included location, time, species and source of ticks, and detection and classification of SFGR.

Quality assessment revealed that the risk of bias scores ranged from 2–3, indicating that all studies were of low risk. The most common risk of bias was the lack of random sample selection. The basic characteristics, extracted data, and quality assessment results of the included studies are summarized in S2 Table.

### 3.3 Spatiotemporal distribution and characteristics of detected ticks and SFGR positive rate

**Fig 2:**
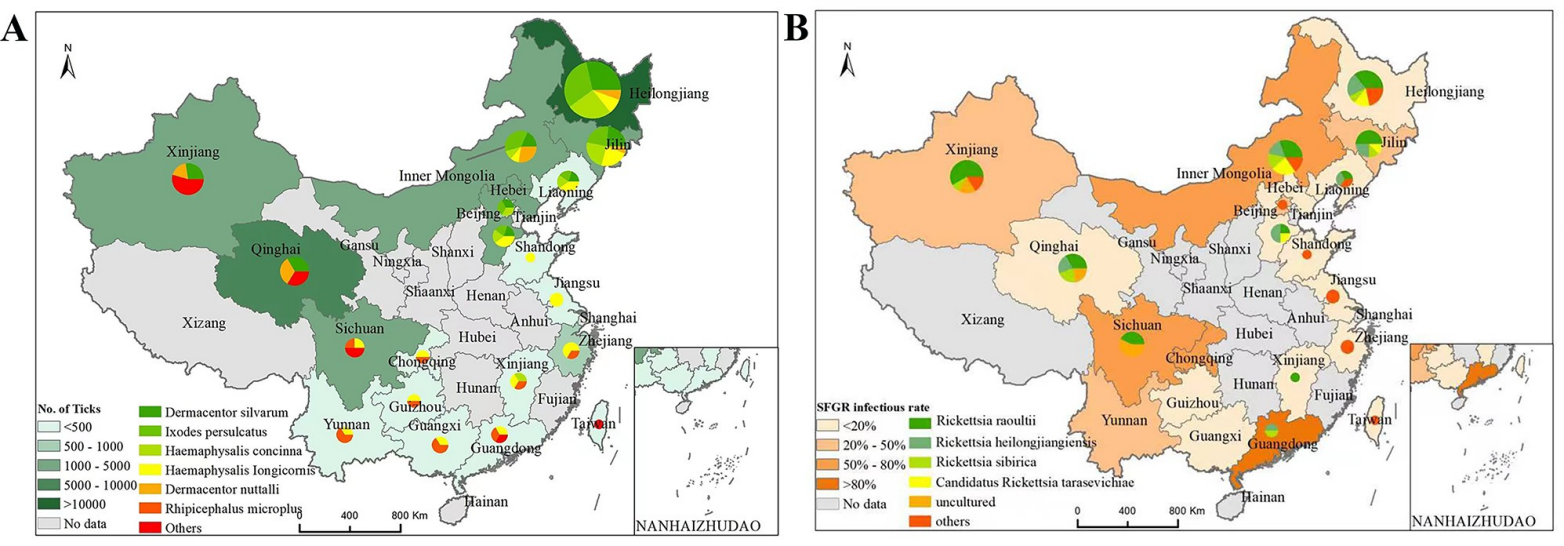
The spatiotemporal distribution of ticks and SFGR in the areas of study of the included literature. (A) Number of studied ticks and tick species classification in different areas; (B) Positive rate and type of SFGR in different regions. Transferred from National Geographic Information Resources Directory Service system (www.webmap.cn).

**Fig 3:**
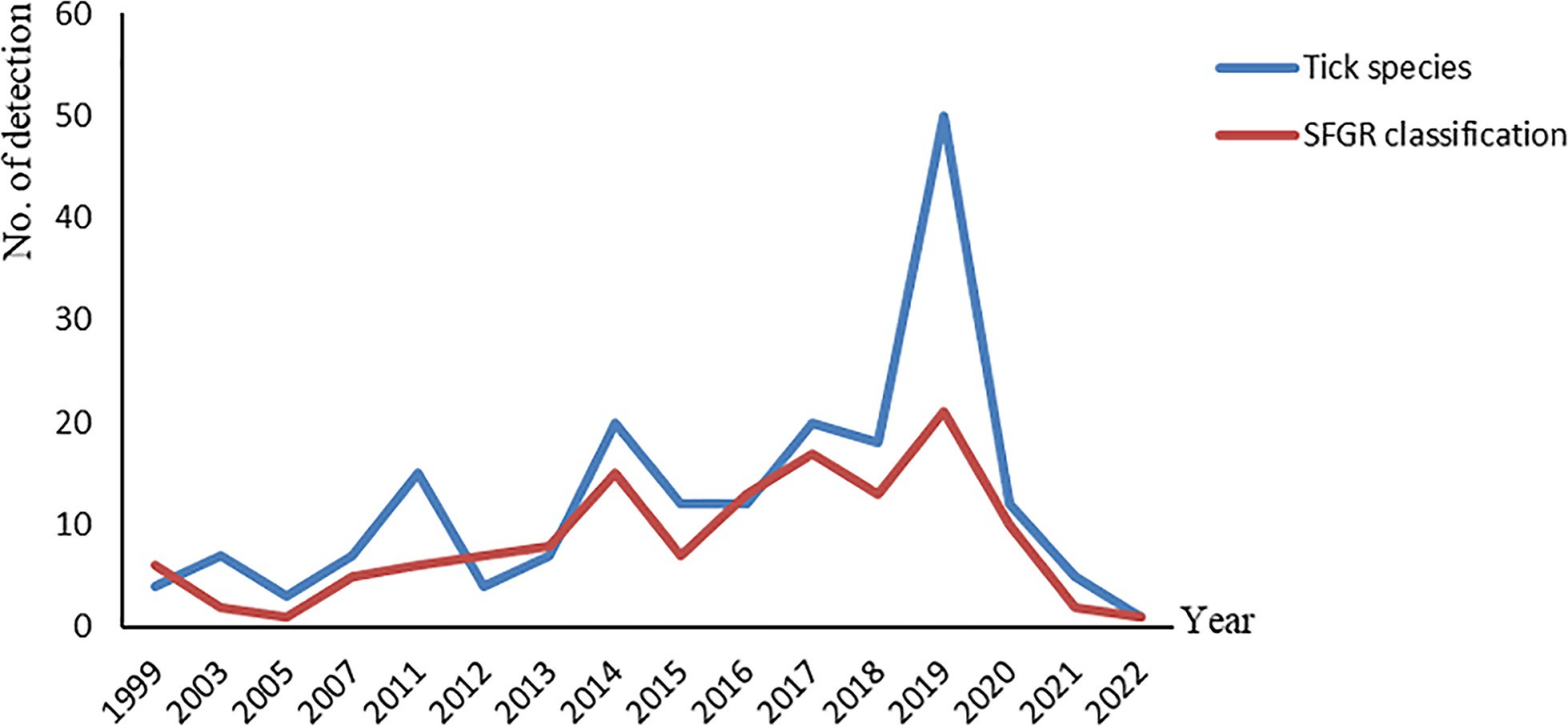
Tick species and SFGR types in studies 2000–2022.

Tick samples in the included studies covered most parts of China and were predominantly obtained from Heilongjiang Province and Qinghai Province (Fig 2A). We found that SFGR positivity was higher in Inner Mongolia Autonomous Region, Chongqing Municipality and Guangdong Province (Fig 2B). The year of publication of the included studies ranged from 2000 to 2022, and the number of tick species and SFGR types identified varied among years (Fig 3). The classification criteria for ticks were reported in 13 articles using stereo microscope. The main morphological classification systems used were Economic Insect Fauna of China (edited by Deng Guofan) and Medical Acariology (edited by Li Chaopin). SFGR was detected by DNA extraction, PCR amplification of the ompA and gltA genes, and 1–1.5% agarose gel electrophoresis.

**Fig 4:**
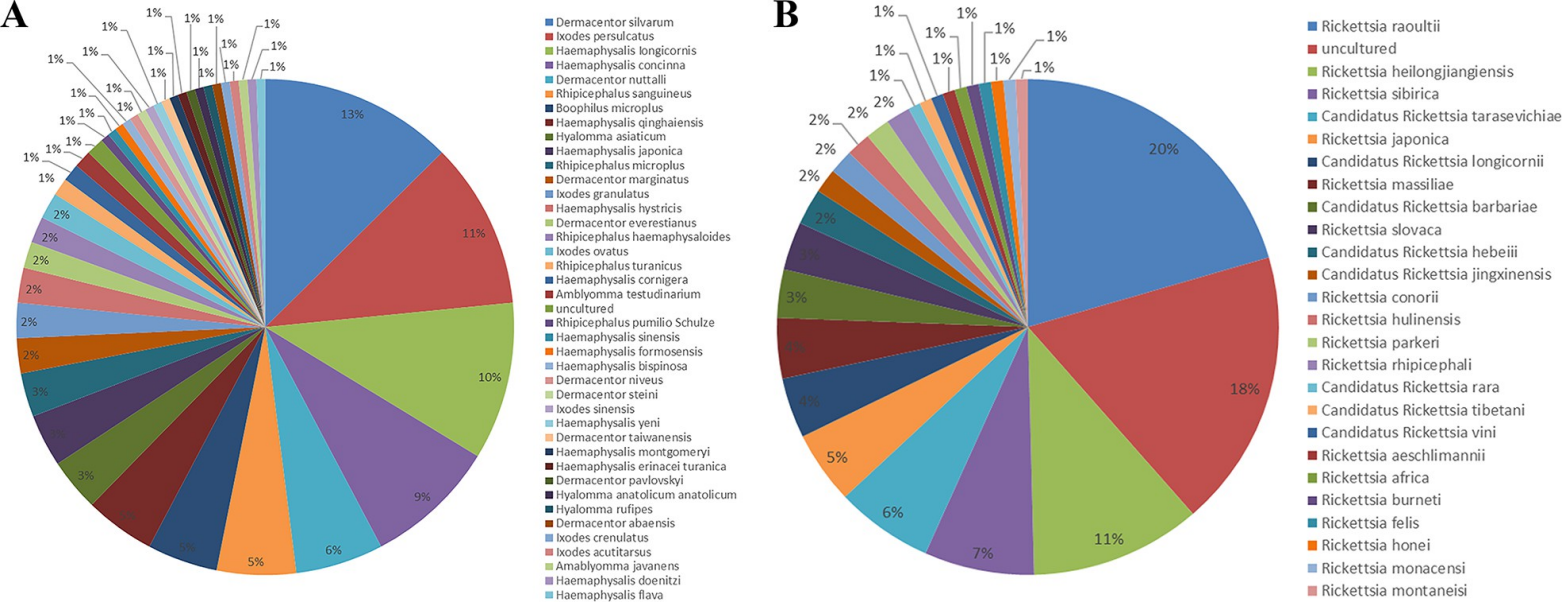
Tick species and SFGR types identified in the 57 included studies. (A) Tick species described in included articles. (B) SFGR types described in included articles.

The 57 studies reported a total of 42 tick species and 25 SFGR types in China. All of the identifiable species belonged to the Ixodidae family (Fig 4). We set the Qinling Mountains-Huaihe River Line as the boundary between northern and southern China, and found that the main sources of ticks consisted of both questing and feeding ticks.

Of 42 tick species identified in the 57 research articles, 21 were in northern China and 26 were in the southern region. In northern cities, the predominant tick species were *Dermacentor silvarum* (19.13%), *Ixodes persulcatus* (16.52%) and *Haemaphysalis concinna* (12.17%), and the main SFGR species were *Rickettsia raoultii* (23.91%) and *Rickettsia heilongiensis* (11.96%). In southern cities, the main tick species were *Haemaphysalis longicornis* (14.55%) and *Rhipicephalus microplus* (9.09%), and the dominant SFGR is currently unculturable (22.86%) (S2 Table).

### 3.4 Meta-analysis of the characteristics of tick species and SFGR positivity

There was a high degree of heterogeneity (*I*^2^ = 99% > 50%, Q-p < 0.001) among the 57 included studies, hence a random effects model was used for meta-analysis.

**Fig 5:**
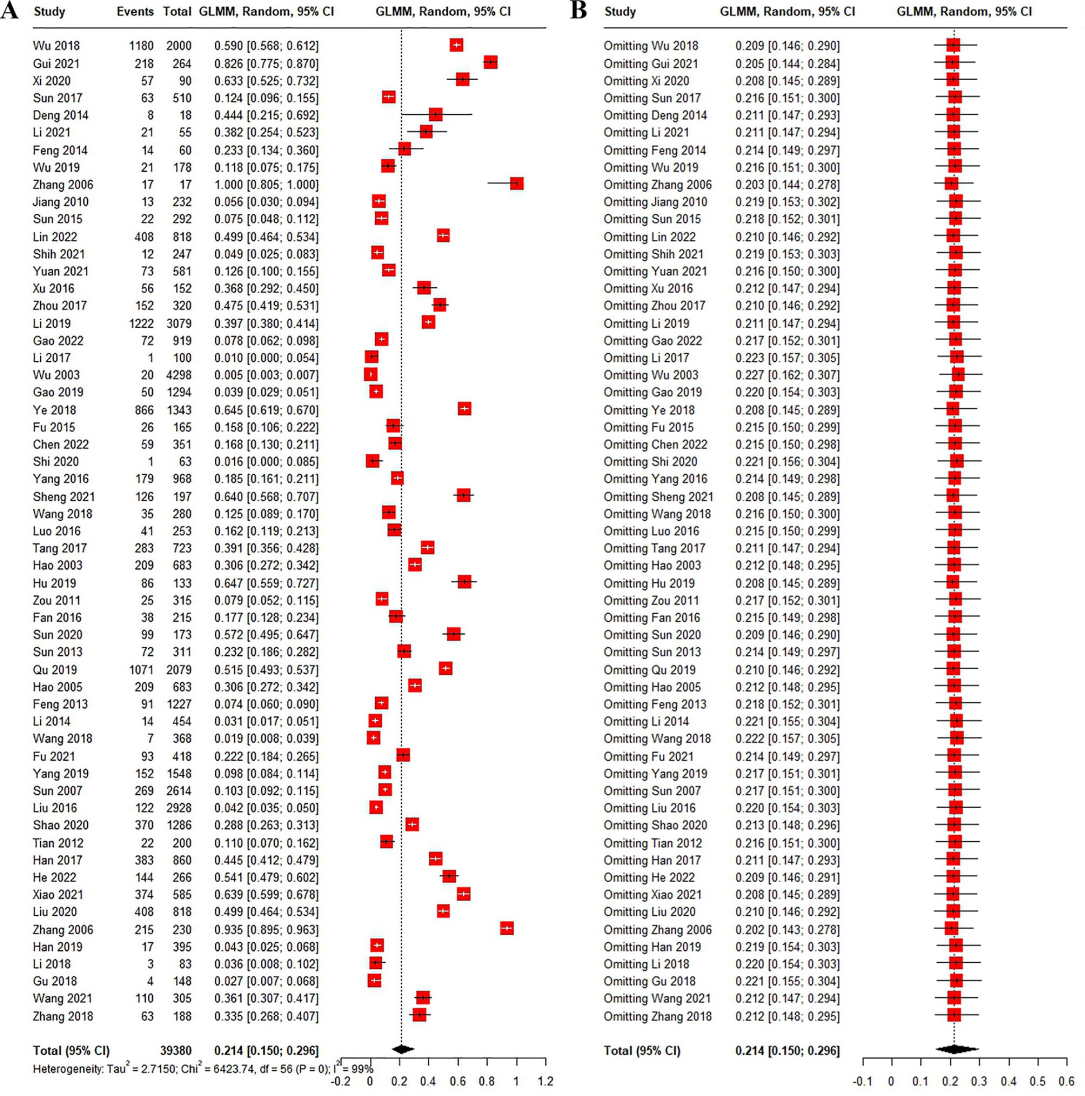
Forest map and Sensitivity analysis of the meta-analysis of 57 included articles. (A) Forest map of tick-borne SFGR positive rate in China. (B) Sensitivity analysis of the meta-analysis of 57 included articles.

**Fig 6:**
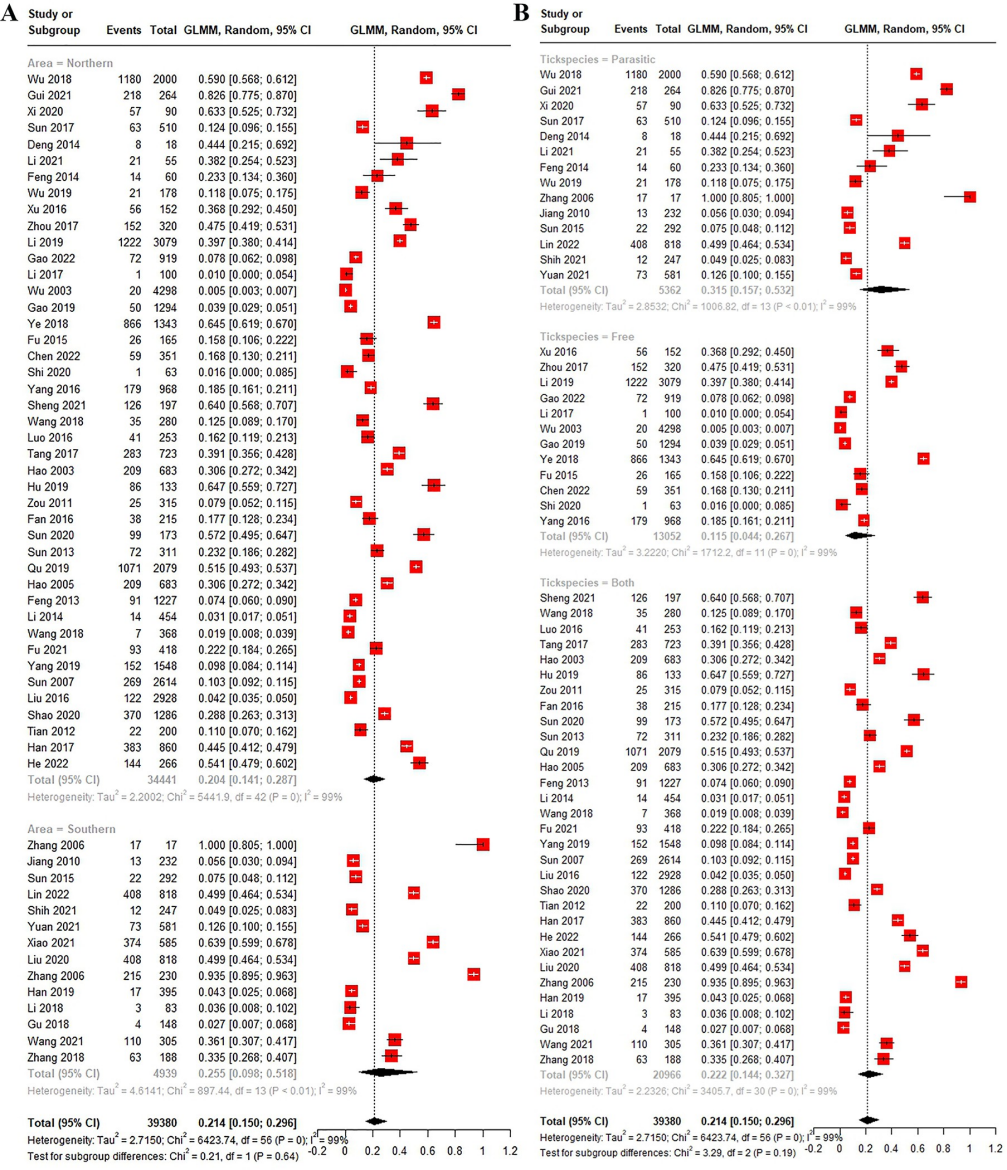
Forest map of tick-borne SFGR positive rate in China based on different subgroups. (A) Forest map of tick-borne SFGR positive rate in China based on different regions. (B) Forest map of tick-borne SFGR positive rate in China based on different tick sources.

Of the 39,380 ticks identified in the 57 studies, the overall positive rate of SFGR was 21.4% (Q = 6423.74, I^2^ = 99%, Q-p < 0.001, 95% CI: 15.0–29.6) (Fig 5A). In terms of region, the positive rate of SFGR was 25.5% (95% CI: 15.0–29.6) in southern China, which was slightly higher than the 20.4% (95% CI: 14.1–28.7) in northern China, but the difference was not statistically significant (Q = 0.21, Q-*p* = 0.64) (Fig 6A). In addition, the positive rate of SFGR in was higher in feeding ticks (31.5%, 95% CI: 15.7–53.2) than in questing ticks (11.5%, 95% CI: 4.4–26.7), but the difference was not significant (Q = 3.29, Q-*p* = 0.19) (Fig 6B).

### 3.5 Sensitivity analysis and publication bias

**Fig 7:**
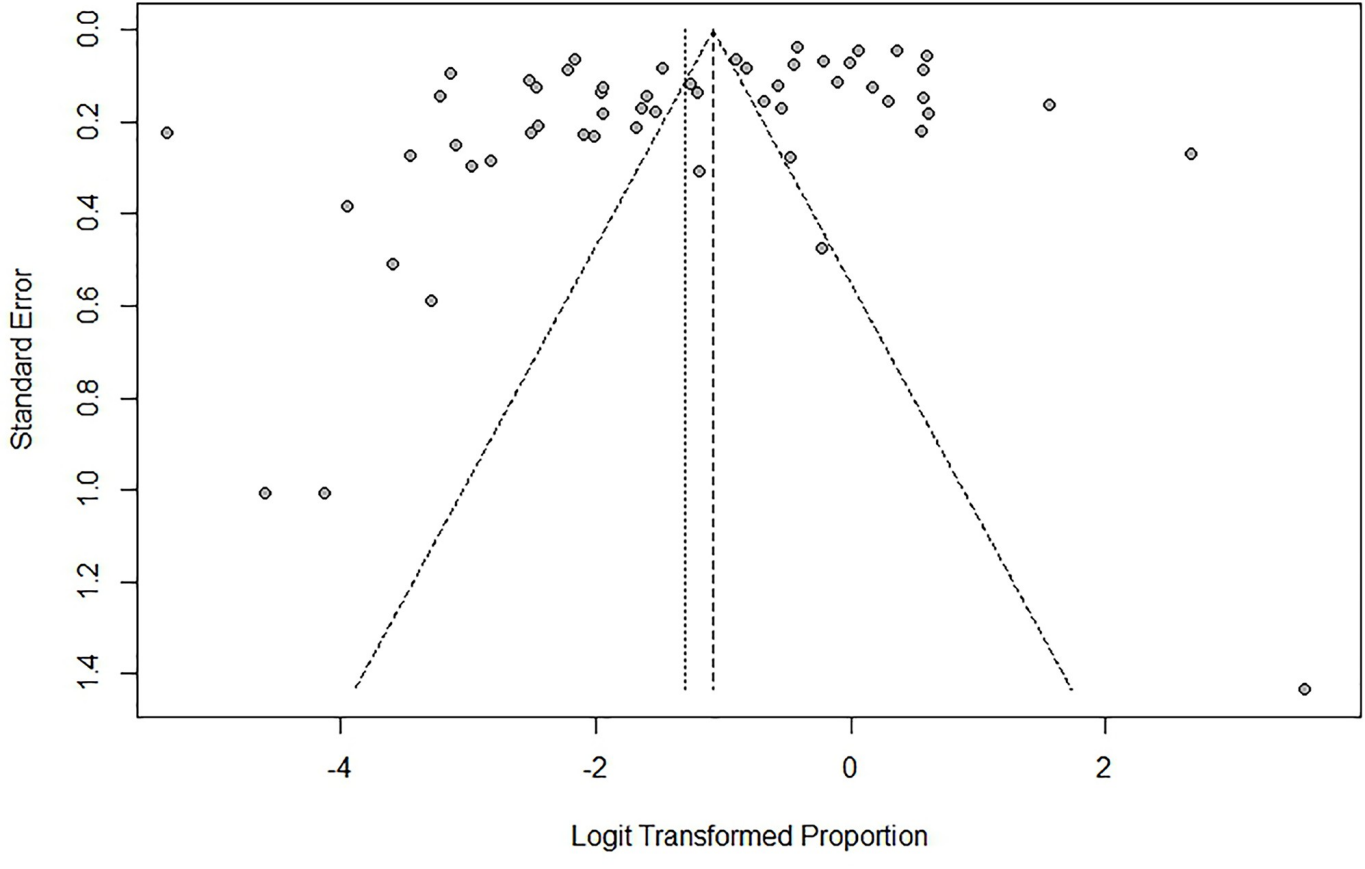
Funnel plot of publication bias in meta-analysis.

Sensitivity analysis demonstrated no significant change in the pooled positive rate after removal of studies and an overlapping 95% CI, indicating good stability of the main results (Fig 5B). Funnel plot and the Egger’s test (*P* = 0.0026 < 0.05) revealed potential publication bias among the studies (Fig 7). Trim and filling method is used to adjust it, with 16 studies added, P = 0.3799 (95%CI: 0.2729–0.5000). There was a significant difference in SGFR positive rate before and after adjustment, suggesting that publication bias may affect the pooled results.

## 4. Discussion

In this study, we identified 39,380 ticks in 57 articles from 8 Chinese and English databases, and SFGR positivity in ticks was 21.4% in China. The sources of ticks included questing ticks and feeding ticks, and the hosts for feeding ticks are cattle, dogs, and goats. Ticks can transmit rickettsia horizontally through bites on mammals [21] and vertically from eggs to offspring, thereby allowing the bacteria to continue circulating in the body and nature [22]. Ticks serve as both the medium and host in SFGR transmission and thus require more stringent control strategies [23].

The distribution characteristics and population abundance of ticks are closely related to their ecological environment. Studies in northern area mainly focus on Northeast China, where the rich ecosystem species and large forest and meadow coverage provide a suitable environment for the survival of ticks. This in turn increases the risk of pathogen transmission via tick bites in host animals and is an important natural epidemic region of tick-borne diseases in China [24]. In this study, the main tick species in northern area was *D*. *silvarum*, which mainly lived in secondary forests, shrublands and forest edge grassland. *D*. *silvarum* is prevalent in Inner Mongolia, Heilongjiang, Xinjiang and other regions of northern China, as well as in Russia and Mongolia [25,26]. Human infections with *R*. *sibirica* and *R*. *heilongjiangensis* have been reported in these area, suggesting that there may be a diverse genotypes in the spotted fever group. Spring and summer are the peak periods of tick activity, and forestry farm workers, agricultural workers and forest sightseeing tourists become susceptible to tick bites [27]. While many new tick-borne Rickettsia species have been discovered in the northern region, very few were reported in the southern region, where the climate and environment are suitable for tick survival and tick-borne disease transmission. In this study, *Haemaphysalis longicornis*, the main tick species in southern China, mainly lived in temperate secondary forests, mountains and hilly marginal areas, while *Rhipicephalus microplus* was predominantly found in grass and bushes in agricultural areas. In the southern region, warmer climates not only prolong the duration of tick activity but also advance the peak period of tick activity from July to May. Tick-borne pathogens are acquired from an infected host and successfully spread to a new host when tickets enter into their next active phase of feeding, which indicates that surveillance work is important for agriculture, animal husbandry, and other risk groups [28].

Spotted fever group of rickettsia is a class of pathogenic microorganisms with obligate intracellular bacteria that causes a variety of natural infectious diseases in humans and animals with fever as the main symptom [29], such as Rocky Mountain spotted fever in the United States [3, 30], Mediterranean spotted fever in Europe, Africa and parts of Asia, and North Asian tick spot fever, Heilongjiang spot fever and Inner Mongolia spot fever in China [2,31,32]. SFGR patients develop long-term specific immunological memory to rickettsia after recovery from infection [33].

The diversity of tick-borne rickettsial diseases is associated with differences in the types of natural foci, with different habitats affecting small mammals such as wild or domestic rodents and the ecosystems of ticks. The main SFGR species in China is *R*. *raoultii*, which is mainly detected in *D*. *silvarum*, *D*. *marginatus* and *D*. *nuttalli*. Studies have shown that *D*. *silvarum* are mainly found on domestic and wild animals in northern China, and are transmitted into susceptible individuals through tick bites and bloodsucking, thus entering the infection cycle. *R*. *raoultii* mainly causes mild to moderate illness in China, which is characterized by fever and fatigue or may also be asymptomatic [34]. This is in contrast to European studies, which have found that *R*. *raoultii* can cause tick-borne lymphadenopathy. *R*. *heilongjiangensis* is the first pathogenic SFGR found in China. It is mainly distributed in Russia and Siberia, and has also been identified in northeast China, Japan and Thailand. *R*. *heilongjiangensis* is a pathogen of tick-borne Far-East spotted fever (FESF) that affects a wide range of hosts, including rodents and mammals, and is widely distributed in both northern and southern regions of China. Infected individuals develop fever, chills, headache, muscle and joint pain, and macular or papular symptoms [35]. However, currently identified SFGRs may only represent the tip of the iceberg when compared to the number of unknown species.

Several limitations in our study should be acknowledged. One major limitation is the uneven distribution of studies across different areas in China. The studies included in this study did not cover all the provinces, cities and autonomous regions in China, and most of them were concentrated in the northern region, which has many forests and animal husbandry development areas. Such uneven distribution may lead to overestimation of SFGR infection rate. Furthermore, publication bias may affect the overall conclusion of our study and care should be taken when interpreting the results. The usefulness of our data in driving public health policy is limited by the lack of overall and severity of clinical burden and accurate representation of variations in risk.

Nonetheless, our study provides valuable insights into the spatiotemporal distribution and biogeographical patterns of tick-borne SFGR infections across China. By acknowledging these limitations, researchers and readers can have a more comprehensive understanding of the potential implications of these findings.

## 5. Conclusion

This study provides key insights into the distribution of tick-borne SFGR infection in China. The overall tick-borne SFGR positive rate is 21.4%, with 20.4% in northern regions and 25.5% in the southern regions. Although most studies of tick-borne SFGR infection rate were conducted in forest areas and developed animal husbandry areas in northern China, there were more tick species in the southern region, highlighting the role of environmental factors such as temperature and precipitation in shaping tick distribution. Successful isolation and stable cultures of rickettsia are challenging, and the research and exploration of pathogenesis is dependent on the availability of stable pathogen isolates. Therefore, strengthening the isolation and identification of new rickettsia strains become an urgent task. Additionally, tick-borne SFGR has a low fatality rate in China and often occurs in remote mountainous areas with limited medical and health resources, leading to insufficient awareness and attention. Since previous studies were mainly molecular epidemiological and serological investigations carried out in different areas, further research combining epidemiological study and high-quality original studies are warranted to analyze SFGR from the population perspective.

Our findings can help raise awareness of tick-borne SFGR diseases among epidemiologists, clinicians, public health officials, and general public in endemic areas. In order to mitigate the risk of public health transmission of tick-borne epidemics, systematic research and comprehensive prevention and control of tick-borne diseases, such as vaccination programs and public awareness campaigns, are warranted.

## Supporting information

S1 Table Search strategies and results by database. (DOCX)

S2 Table Studies included in systematic literature review contributing to tick-borne SFGR positive rate analyses. (DOCX)

S3 Table PRISMA 2020 checklist. (DOCX)

## References

[1] Cohen R, Finn T, Babushkin F, Paran Y, Ben Ami R, Atamna A, et al. Spotted Fever Group Rickettsioses in Israel, 2010–2019. Emerg Infect Dis. 2021;27(8): 2117–2126. doi: 10.3201/eid2708.203661 34286684 PMC8314820

[2] Gu XL, Wang R, Zhou CM, Cui JT, Li ZM, Jiang ZZ, et al. Natural Mediterranean Spotted Fever Foci, Qingdao, China. Emerg Infect Dis. 2022;28(12): 2524–2527. doi: 10.3201/eid2812.221097 36417960 PMC9707604

[3] Zazueta OE, Armstrong PA, Márquez-Elguea A, Hernández Milán NS, Peterson AE, Ovalle-Marroquín DF, et al. Rocky Mountain Spotted Fever in a Large Metropolitan Center, Mexico-United States Border, 2009–2019. Emerg Infect Dis. 2021;27(6): 1567–1576. doi: 10.3201/eid2706.191662 34014151 PMC8153879

[4] Cheng C, Fu W, Ju W, Yang L, Xu N, Wang YM, et al. Diversity of spotted fever group Rickettsia infection in hard ticks from Suifenhe, Chinese-Russian border. Ticks Tick Borne Dis. 2016;7(5): 715–719. doi: 10.1016/j.ttbdis.2016.02.023 26976703

[5] Han J, He Z, Shao Z. Research progress of common tick-borne Rickettsia. Chinese Journal of Hygienic Insecticides & Equipments. 2022;28(01): 86–89.

[6] Jensen BB, Bruun MT, Jensen PM, Pedersen AK, Fournier PE, Skarphedinsson S, et al. Evaluation of factors influencing tick bites and tick-borne infections: a longitudinal study. Parasit Vectors. 2021;14(1): 289. doi: 10.1186/s13071-021-04751-0 34051820 PMC8164064

[7] Lkhagvatseren S, Hogan KM, Boldbaatar B, von Fricken ME, Anderson BD, Pulscher LA, et al. Discrepancies between self-reported tick bites and evidence of tick-borne disease exposure among nomadic Mongolian herders. Zoonoses Public Health. 2019;66(5): 480–486. doi: 10.1111/zph.12579 30969028 PMC6629472

[8] Sheng Y, Deng H, Gao Y, Li Y, Zhu J, Niu T, et al. A survey of tick-carried spotted fever group Rickettsia in Inner Mongolia from 2019 to 2020. Chinese Journal of Frontier Health and Quarantine. 2021;44(03): 168–170.

[9] Teng Z, Zhao N, Ren R, Zhang X, Du Z, Wang P, et al. Human Rickettsia felis infections in Mainland China. Front Cell Infect Microbiol. 2022;12: 997315. doi: 10.3389/fcimb.2022.997315 36211956 PMC9537614

[10] Martello E, Mannelli A, Grego E, Ceballos LA, Ragagli C, Stella MC, et al. Borrelia burgdorferi sensu lato and spotted fever group rickettsiae in small rodents and attached ticks in the Northern Apennines, Italy. Ticks Tick Borne Dis. 2019;10(4): 862–867. doi: 10.1016/j.ttbdis.2019.04.005 31014939

[11] Wong KH, Shapiro ED, Soffer GK. A Review of Post-treatment Lyme Disease Syndrome and Chronic Lyme Disease for the Practicing Immunologist. Clin Rev Allergy Immunol. 2022;62(1): 264–271. doi: 10.1007/s12016-021-08906-w 34687445

[12] Bloch EM, Kumar S, Krause PJ. Persistence of Babesia microti Infection in Humans. Pathogens. 2019;8(3): doi: 10.3390/pathogens8030102 31319461 PMC6789900

[13] Efstratiou A, Karanis G, Karanis P. Tick-Borne Pathogens and Diseases in Greece. Microorganisms. 2021;9(8): doi: 10.3390/microorganisms9081732 34442811 PMC8399993

[14] Díaz-Sánchez AA, Chilton NB, Roblejo-Arias L, Fonseca-Rodríguez O, Marrero-Perera R, Diyes CP, et al. Molecular detection and identification of spotted fever group rickettsiae in ticks collected from horses in Cuba. Med Vet Entomol. 2021;35(2): 207–212. doi: 10.1111/mve.12480 32936461

[15] Kim HK. Rickettsia-Host-Tick Interactions: Knowledge Advances and Gaps. Infect Immun. 2022;90(9): e0062121. doi: 10.1128/iai.00621-21 35993770 PMC9476906

[16] Wilson JM, Breitschwerdt EB, Juhasz NB, Marr HS, de Brito Galvão JF, Pratt CL, et al. Novel Rickettsia Species Infecting Dogs, United States. Emerg Infect Dis. 2020;26(12): 3011–3015. doi: 10.3201/eid2612.200272 33219793 PMC7706976

[17] Zhao GP, Wang YX, Fan ZW, Ji Y, Liu MJ, Zhang WH, et al. Mapping ticks and tick-borne pathogens in China. Nat Commun. 2021;12(1): 1075. doi: 10.1038/s41467-021-21375-1 33597544 PMC7889899

[18] Xing Y, Schmitt HJ, Arguedas A, Yang J. Tick-borne encephalitis in China: A review of epidemiology and vaccines. Vaccine. 2017;35(9): 1227–1237. doi: 10.1016/j.vaccine.2017.01.015 28153343

[19] Sands BO, Bryer KE, Wall R. Climate and the seasonal abundance of the tick Dermacentor reticulatus. Med Vet Entomol. 2021;35(3): 434–441. doi: 10.1111/mve.12518 33942903

[20] Hoy D, Brooks P, Woolf A, Blyth F, March L, Bain C, et al. Assessing risk of bias in prevalence studies: modification of an existing tool and evidence of interrater agreement. J Clin Epidemiol. 2012;65(9): 934–939. doi: 10.1016/j.jclinepi.2011.11.014 22742910

[21] Guerrib F, Ning C, Mateos-Hernandéz L, Rakotobe S, Park Y, Hajdusek O, et al. Dual SIFamide receptors in Ixodes salivary glands. Insect Biochem Mol Biol. 2023;158: 103963. doi: 10.1016/j.ibmb.2023.103963 37257628

[22] Ogata S, Umemiya-Shirafuji R, Kusakisako K, Kakisaka K, Chatanga E, Hayashi N, et al. Investigation of vertical and horizontal transmission of Spiroplasma in ticks under laboratory conditions. Sci Rep. 2023;13(1): 13265. doi: 10.1038/s41598-023-39128-z 37582809 PMC10427632

[23] Ketsarin Kamyingkird MZS-A, Mohamed Abdo Rizk. Editorial: Treatment of tick-borne diseases: current status, challenges, and global perspectives. Frontiers in Pharmacology. 2024;15:1366988:38533253 10.3389/fphar.2024.1366988 PMC10963605

[24] Yang Li YB, Wenli Liu, Jing Li, Fengjuan Tian, Xiaohu Han, Lei Liu, Yigang Tong. Diversity analysis of tick-associated viruses in northeast China. Virologica Sinica. 2023;38(6): 961–965. doi: 10.1016/j.virs.2023.10.003 37832718 PMC10786652

[25] Jiang BG, Jia N, Jiang JF, Zheng YC, Chu YL, Jiang RR, et al. Borrelia miyamotoi Infections in Humans and Ticks, Northeastern China. Emerg Infect Dis. 2018;24(2): 236–241. doi: 10.3201/eid2402.160378 29350133 PMC5782893

[26] Maschalidi S, Mehrotra P, Keceli BN, De Cleene HKL, Lecomte K, Van der Cruyssen R, et al. Targeting SLC7A11 improves efferocytosis by dendritic cells and wound healing in diabetes. Nature. 2022;606(7915): 776–784. doi: 10.1038/s41586-022-04754-6 35614212

[27] Guo-Ping Zhao Y-XW, Zheng-Wei Fan, Yang Ji, Ming-Jin Liu, Wen-Hui Zhang, Xin-Lou Li, Shi-Xia Zhou, Hao Li, Song Liang, Wei Liu, Yang Yang, Li-Qun Fang Mapping ticks and tick-borne pathogens in China. Nature Communication. 2021;12(1): 1075. doi: 10.1038/s41467-021-21375-1 33597544 PMC7889899

[28] El-Sayed El-Alfy IA, Somaya Saleh, Rana Elseadawy, Fereig Ragab M, Mohamed Abdo Rizk, Xuenan Xuan. Tick-borne pathogens in camels: A systematic review and meta-analysis of the prevalence in dromedaries. Ticks and Tick-borne Diseases. 2024;15(1): 102268. doi: 10.1016/j.ttbdis.2023.102268 37769585

[29] McCall CL, Curns AT, Rotz LD, Singleton JA Jr., Treadwell TA, Comer JA, et al. Fort Chaffee revisited: the epidemiology of tick-borne rickettsial and ehrlichial diseases at a natural focus. Vector Borne Zoonotic Dis. 2001;1(2): 119–127. doi: 10.1089/153036601316977723 12653142

[30] Kidd L. Emerging Spotted Fever Rickettsioses in the United States. Vet Clin North Am Small Anim Pract. 2022;52(6): 1305–1317. doi: 10.1016/j.cvsm.2022.07.003 36336422

[31] Gomez-Barroso D, Vescio MF, Bella A, Ciervo A, Busani L, Rizzo C, et al. Mediterranean spotted fever rickettsiosis in Italy, 2001–2015: Spatio-temporal distribution based on hospitalization records. Ticks Tick Borne Dis. 2019;10(1): 43–50. doi: 10.1016/j.ttbdis.2018.09.001 30197269

[32] Eneku W, Erima B, Byaruhanga AM, Atim G, Tugume T, Ukuli QA, et al. Wide distribution of Mediterranean and African spotted fever agents and the first identification of Israeli spotted fever agent in ticks in Uganda. PLoS Negl Trop Dis. 2023;17(7): e0011273. doi: 10.1371/journal.pntd.0011273 37498943 PMC10409254

[33] Sonenshine DE, Macaluso KR. Microbial Invasion vs. Tick Immune Regulation. Front Cell Infect Microbiol. 2017;7: 390. doi: 10.3389/fcimb.2017.00390 28929088 PMC5591838

[34] Li H, Zhang PH, Huang Y, Du J, Cui N, Yang ZD, et al. Isolation and Identification of Rickettsia raoultii in Human Cases: A Surveillance Study in 3 Medical Centers in China. Clin Infect Dis. 2018;66(7): 1109–1115. doi: 10.1093/cid/cix917 29069294

[35] Mediannikov OY, Sidelnikov Y, Ivanov L, Mokretsova E, Fournier PE, Tarasevich I, et al. Acute tick-borne rickettsiosis caused by Rickettsia heilongjiangensis in Russian Far East. Emerg Infect Dis. 2004;10(5): 810–817. doi: 10.3201/eid1005.030437 15200813 PMC3323216

