# Supplementary material for: A systematic review and meta-analysis of the prevalence of tick-borne SFGR in China from 2000 to 2022
Source: PLoS Negl Trop Dis. 2024 Oct 9;18(10):e0012550. doi: 10.1371/journal.pntd.0012550 (PMC11463837; doi:10.1371/journal.pntd.0012550)
Supplement: S1 Table — (DOCX) [file pntd.0012550.s001.docx]

**S1 Table. Search strategies and results by database.**

| **Database** | **Search strategy** | **Results** |
| --- | --- | --- |
| PubMed | 1 "Ticks"[MeSH Terms] 23,715  2 "ticks"[All Fields] OR "ticks"[MeSH Terms] OR "ticks"[All Fields] OR ("ticks"[MeSH Terms] OR "ticks"[All Fields] OR "tick"[All Fields]) OR ("ixodidae"[MeSH Terms] OR "ixodidae"[All Fields] OR "ticks"[MeSH Terms] OR "ticks"[All Fields] OR "ixodida"[All Fields]) OR ("ticks"[MeSH Terms] OR "ticks"[All Fields]) OR ("ixodidae"[MeSH Terms] OR "ixodidae"[All Fields] OR "ticks"[MeSH Terms] OR "ticks"[All Fields] OR "ixodida"[All Fields]) OR ("argasidae"[MeSH Terms] OR "argasidae"[All Fields]) OR ("argas"[MeSH Terms] OR "argas"[All Fields]) OR ("ornithodoros"[MeSH Terms] OR "ornithodoros"[All Fields]) OR ("ixodidae"[MeSH Terms] OR "ixodidae"[All Fields] OR "ticks"[MeSH Terms] OR "ticks"[All Fields] OR "ixodida"[All Fields]) OR ("amblyomma"[MeSH Terms] OR "amblyomma"[All Fields]) OR ("dermacentor"[MeSH Terms] OR "dermacentor"[All Fields]) OR ("ixodes"[MeSH Terms] OR "ixodes"[All Fields] OR "ixode"[All Fields] OR "ixodic"[All Fields]) OR ("rhipicephalus"[MeSH Terms] OR "rhipicephalus"[All Fields]) OR "Ixodoidea"[All Fields] OR ("vector borne diseases"[MeSH Terms] OR ("vector"[All Fields] AND "borne"[All Fields] AND "diseases"[All Fields]) OR "vector borne diseases"[All Fields]) OR ("vector borne diseases"[MeSH Terms] OR ("vector"[All Fields] AND "borne"[All Fields] AND "diseases"[All Fields]) OR "vector borne diseases"[All Fields]) OR ("vector borne diseases"[MeSH Terms] OR ("vector"[All Fields] AND "borne"[All Fields] AND "diseases"[All Fields]) OR "vector borne diseases"[All Fields] OR ("vector"[All Fields] AND "borne"[All Fields] AND "disease"[All Fields]) OR "vector borne disease"[All Fields]) OR ("vector borne diseases"[MeSH Terms] OR ("vector"[All Fields] AND "borne"[All Fields] AND "diseases"[All Fields]) OR "vector borne diseases"[All Fields] OR ("vectorborne"[All Fields] AND "diseases"[All Fields]) OR "vectorborne diseases"[All Fields]) OR ("vector borne diseases"[MeSH Terms] OR ("vector"[All Fields] AND "borne"[All Fields] AND "diseases"[All Fields]) OR "vector borne diseases"[All Fields] OR ("vectorborne"[All Fields] AND "disease"[All Fields]) OR "vectorborne disease"[All Fields]) OR ("vector borne diseases"[MeSH Terms] OR ("vector"[All Fields] AND "borne"[All Fields] AND "diseases"[All Fields]) OR "vector borne diseases"[All Fields] OR ("mosquito"[All Fields] AND "borne"[All Fields] AND "diseases"[All Fields]) OR "mosquito borne diseases"[All Fields]) OR ("vector borne diseases"[MeSH Terms] OR ("vector"[All Fields] AND "borne"[All Fields] AND "diseases"[All Fields]) OR "vector borne diseases"[All Fields] OR ("mosquito"[All Fields] AND "borne"[All Fields] AND "disease"[All Fields]) OR "mosquito borne disease"[All Fields]) OR ("vector borne diseases"[MeSH Terms] OR ("vector"[All Fields] AND "borne"[All Fields] AND "diseases"[All Fields]) OR "vector borne diseases"[All Fields] OR ("mosquito"[All Fields] AND "borne"[All Fields] AND "diseases"[All Fields]) OR "mosquito borne diseases"[All Fields]) OR ("vector borne diseases"[MeSH Terms] OR ("vector"[All Fields] AND "borne"[All Fields] AND "diseases"[All Fields]) OR "vector borne diseases"[All Fields] OR ("mosquito"[All Fields] AND "borne"[All Fields] AND "disease"[All Fields]) OR "mosquito borne disease"[All Fields]) OR ("tick borne diseases"[MeSH Terms] OR ("tick borne"[All Fields] AND "diseases"[All Fields]) OR "tick borne diseases"[All Fields] OR ("tick"[All Fields] AND "borne"[All Fields] AND "diseases"[All Fields]) OR "tick borne diseases"[All Fields]) OR ("african swine fever"[MeSH Terms] OR ("african"[All Fields] AND "swine"[All Fields] AND "fever"[All Fields]) OR "african swine fever"[All Fields]) OR ("anaplasmosis"[MeSH Terms] OR "anaplasmosis"[All Fields] OR "anaplasmoses"[All Fields]) OR ("babesiosis"[MeSH Terms] OR "babesiosis"[All Fields] OR "babesioses"[All Fields]) OR ("colorado tick fever"[MeSH Terms] OR ("colorado"[All Fields] AND "tick"[All Fields] AND "fever"[All Fields]) OR "colorado tick fever"[All Fields]) OR ("ehrlichiosis"[MeSH Terms] OR "ehrlichiosis"[All Fields] OR "ehrlichioses"[All Fields]) OR ("encephalitis, tick borne"[MeSH Terms] OR ("encephalitis"[All Fields] AND "tick borne"[All Fields]) OR "tick-borne encephalitis"[All Fields] OR ("encephalitis"[All Fields] AND "tick"[All Fields] AND "borne"[All Fields]) OR "encephalitis tick borne"[All Fields]) OR ("hemorrhagic fever, crimean"[MeSH Terms] OR ("hemorrhagic"[All Fields] AND "fever"[All Fields] AND "crimean"[All Fields]) OR "crimean hemorrhagic fever"[All Fields] OR ("hemorrhagic"[All Fields] AND "fever"[All Fields] AND "crimean"[All Fields]) OR "hemorrhagic fever crimean"[All Fields]) OR ("hemorrhagic fever, omsk"[MeSH Terms] OR ("hemorrhagic"[All Fields] AND "fever"[All Fields] AND "omsk"[All Fields]) OR "omsk hemorrhagic fever"[All Fields] OR ("hemorrhagic"[All Fields] AND "fever"[All Fields] AND "omsk"[All Fields]) OR "hemorrhagic fever omsk"[All Fields]) OR ("kyasanur forest disease"[MeSH Terms] OR ("kyasanur"[All Fields] AND "forest"[All Fields] AND "disease"[All Fields]) OR "kyasanur forest disease"[All Fields]) OR ("lyme disease"[MeSH Terms] OR ("lyme"[All Fields] AND "disease"[All Fields]) OR "lyme disease"[All Fields]) OR ("nairobi sheep disease"[MeSH Terms] OR ("nairobi"[All Fields] AND "sheep"[All Fields] AND "disease"[All Fields]) OR "nairobi sheep disease"[All Fields]) OR ("relapsing fever"[MeSH Terms] OR ("relapsing"[All Fields] AND "fever"[All Fields]) OR "relapsing fever"[All Fields]) OR ("severe fever with thrombocytopenia syndrome"[MeSH Terms] OR ("severe"[All Fields] AND "fever"[All Fields] AND "thrombocytopenia"[All Fields] AND "syndrome"[All Fields]) OR "severe fever with thrombocytopenia syndrome"[All Fields]) OR ("spotted fever group rickettsiosis"[MeSH Terms] OR ("spotted"[All Fields] AND "fever"[All Fields] AND "group"[All Fields] AND "rickettsiosis"[All Fields]) OR "spotted fever group rickettsiosis"[All Fields]) OR ("theileriasis"[MeSH Terms] OR "theileriasis"[All Fields]) OR ("tularaemia"[All Fields] OR "tularemia"[MeSH Terms] OR "tularemia"[All Fields]) OR (("bacterial"[All Fields] OR "bacterially"[All Fields] OR "bacterials"[All Fields]) AND ("genetic vectors"[MeSH Terms] OR ("genetic"[All Fields] AND "vectors"[All Fields]) OR "genetic vectors"[All Fields] OR "vector"[All Fields] OR "vectors"[All Fields] OR "vector s"[All Fields] OR "vectored"[All Fields] OR "vectoring"[All Fields] OR "vectorization"[All Fields] OR "vectorize"[All Fields] OR "vectorized"[All Fields] OR "vectorizing"[All Fields])) OR (("bacterial"[All Fields] OR "bacterially"[All Fields] OR "bacterials"[All Fields]) AND ("genetic vectors"[MeSH Terms] OR ("genetic"[All Fields] AND "vectors"[All Fields]) OR "genetic vectors"[All Fields] OR "vector"[All Fields] OR "vectors"[All Fields] OR "vector s"[All Fields] OR "vectored"[All Fields] OR "vectoring"[All Fields] OR "vectorization"[All Fields] OR "vectorize"[All Fields] OR "vectorized"[All Fields] OR "vectorizing"[All Fields])) OR ("arachnid vectors"[MeSH Terms] OR ("arachnid"[All Fields] AND "vectors"[All Fields]) OR "arachnid vectors"[All Fields]) OR ("arachnid vectors"[MeSH Terms] OR ("arachnid"[All Fields] AND "vectors"[All Fields]) OR "arachnid vectors"[All Fields] OR ("arachnid"[All Fields] AND "vector"[All Fields]) OR "arachnid vector"[All Fields]) OR ("arachnid vectors"[MeSH Terms] OR ("arachnid"[All Fields] AND "vectors"[All Fields]) OR "arachnid vectors"[All Fields] OR ("vectors"[All Fields] AND "arachnid"[All Fields])) 306,444  3 "Rickettsia"[MeSH Terms] 6,349  4 "Rickettsia"[MeSH Terms] OR ("rickettsia infections"[MeSH Terms] OR ("Rickettsia"[All Fields] AND "infections"[All Fields]) OR "rickettsia infections"[All Fields] OR ("boutonneuse fever"[MeSH Terms] OR ("boutonneuse"[All Fields] AND "fever"[All Fields]) OR "boutonneuse fever"[All Fields]) OR ("rocky mountain spotted fever"[MeSH Terms] OR ("rocky"[All Fields] AND "mountain"[All Fields] AND "spotted"[All Fields] AND "fever"[All Fields]) OR "rocky mountain spotted fever"[All Fields]) OR ("typhus, endemic flea borne"[MeSH Terms] OR ("typhus"[All Fields] AND "endemic"[All Fields] AND "flea borne"[All Fields]) OR "endemic flea-borne typhus"[All Fields] OR ("typhus"[All Fields] AND "endemic"[All Fields] AND "flea"[All Fields] AND "borne"[All Fields]) OR "typhus endemic flea borne"[All Fields]) OR ("typhus, epidemic louse borne"[MeSH Terms] OR ("typhus"[All Fields] AND "epidemic"[All Fields] AND "louse borne"[All Fields]) OR "epidemic louse-borne typhus"[All Fields] OR ("typhus"[All Fields] AND "epidemic"[All Fields] AND "louse"[All Fields] AND "borne"[All Fields]) OR "typhus epidemic louse borne"[All Fields]) OR ("rickettsia infections"[MeSH Terms] OR ("Rickettsia"[All Fields] AND "infections"[All Fields]) OR "rickettsia infections"[All Fields] OR ("rickettsial"[All Fields] AND "diseases"[All Fields]) OR "rickettsial diseases"[All Fields]) OR ("rickettsia infections"[MeSH Terms] OR ("Rickettsia"[All Fields] AND "infections"[All Fields]) OR "rickettsia infections"[All Fields] OR ("rickettsial"[All Fields] AND "disease"[All Fields]) OR "rickettsial disease"[All Fields]) OR ("rickettsia infections"[MeSH Terms] OR ("Rickettsia"[All Fields] AND "infections"[All Fields]) OR "rickettsia infections"[All Fields] OR "rickettsiosis"[All Fields]) OR ("rickettsia infections"[MeSH Terms] OR ("Rickettsia"[All Fields] AND "infections"[All Fields]) OR "rickettsia infections"[All Fields] OR "rickettsioses"[All Fields]) OR (("Rickettsia"[MeSH Terms] OR "Rickettsia"[All Fields] OR "rickettsiae"[All Fields] OR "rickettsiales"[MeSH Terms] OR "rickettsiales"[All Fields] OR "rickettsias"[All Fields]) AND ("infectious"[All Fields] OR "infectiousness"[All Fields])) OR ("rickettsia infections"[MeSH Terms] OR ("Rickettsia"[All Fields] AND "infections"[All Fields]) OR "rickettsia infections"[All Fields] OR ("Rickettsia"[All Fields] AND "infection"[All Fields]) OR "rickettsia infection"[All Fields]) OR ("spotted fever group rickettsiosis"[MeSH Terms] OR ("spotted"[All Fields] AND "fever"[All Fields] AND "group"[All Fields] AND "rickettsiosis"[All Fields]) OR "spotted fever group rickettsiosis"[All Fields]) OR ("spotted fever group rickettsiosis"[MeSH Terms] OR ("spotted"[All Fields] AND "fever"[All Fields] AND "group"[All Fields] AND "rickettsiosis"[All Fields]) OR "spotted fever group rickettsiosis"[All Fields] OR ("spotted"[All Fields] AND "fevers"[All Fields]) OR "spotted fevers"[All Fields]) OR ("spotted fever group rickettsiosis"[MeSH Terms] OR ("spotted"[All Fields] AND "fever"[All Fields] AND "group"[All Fields] AND "rickettsiosis"[All Fields]) OR "spotted fever group rickettsiosis"[All Fields] OR ("spotted"[All Fields] AND "fever"[All Fields]) OR "spotted fever"[All Fields]) OR ("spotted fever group rickettsiosis"[MeSH Terms] OR ("spotted"[All Fields] AND "fever"[All Fields] AND "group"[All Fields] AND "rickettsiosis"[All Fields]) OR "spotted fever group rickettsiosis"[All Fields] OR ("tick"[All Fields] AND "borne"[All Fields] AND "lymphadenopathy"[All Fields]) OR "tick borne lymphadenopathy"[All Fields]) OR ("spotted fever group rickettsiosis"[MeSH Terms] OR ("spotted"[All Fields] AND "fever"[All Fields] AND "group"[All Fields] AND "rickettsiosis"[All Fields]) OR "spotted fever group rickettsiosis"[All Fields] OR ("tick"[All Fields] AND "borne"[All Fields] AND "lymphadenopathy"[All Fields]) OR "tick borne lymphadenopathy"[All Fields]) OR ("spotted fever group rickettsiosis"[MeSH Terms] OR ("spotted"[All Fields] AND "fever"[All Fields] AND "group"[All Fields] AND "rickettsiosis"[All Fields]) OR "spotted fever group rickettsiosis"[All Fields] OR ("tick"[All Fields] AND "borne"[All Fields] AND "lymphadenopathies"[All Fields])) OR ("spotted fever group rickettsiosis"[MeSH Terms] OR ("spotted"[All Fields] AND "fever"[All Fields] AND "group"[All Fields] AND "rickettsiosis"[All Fields]) OR "spotted fever group rickettsiosis"[All Fields] OR ("Rickettsia"[All Fields] AND "slovaca"[All Fields] AND "infections"[All Fields]) OR "rickettsia slovaca infections"[All Fields]) OR ("spotted fever group rickettsiosis"[MeSH Terms] OR ("spotted"[All Fields] AND "fever"[All Fields] AND "group"[All Fields] AND "rickettsiosis"[All Fields]) OR "spotted fever group rickettsiosis"[All Fields] OR "tibola"[All Fields]) OR ("spotted fever group rickettsiosis"[MeSH Terms] OR ("spotted"[All Fields] AND "fever"[All Fields] AND "group"[All Fields] AND "rickettsiosis"[All Fields]) OR "spotted fever group rickettsiosis"[All Fields] OR ("african"[All Fields] AND "tick"[All Fields] AND "bite"[All Fields] AND "fever"[All Fields]) OR "african tick bite fever"[All Fields]) OR ("spotted fever group rickettsiosis"[MeSH Terms] OR ("spotted"[All Fields] AND "fever"[All Fields] AND "group"[All Fields] AND "rickettsiosis"[All Fields]) OR "spotted fever group rickettsiosis"[All Fields] OR ("african"[All Fields] AND "tick"[All Fields] AND "bite"[All Fields] AND "fever"[All Fields]) OR "african tick bite fever"[All Fields]) OR ("spotted fever group rickettsiosis"[MeSH Terms] OR ("spotted"[All Fields] AND "fever"[All Fields] AND "group"[All Fields] AND "rickettsiosis"[All Fields]) OR "spotted fever group rickettsiosis"[All Fields] OR ("flinders"[All Fields] AND "island"[All Fields] AND "spotted"[All Fields] AND "fever"[All Fields]) OR "flinders island spotted fever"[All Fields]) OR (("spotted fever group rickettsiosis"[MeSH Terms] OR ("spotted"[All Fields] AND "fever"[All Fields] AND "group"[All Fields] AND "rickettsiosis"[All Fields]) OR "spotted fever group rickettsiosis"[All Fields] OR ("spotted"[All Fields] AND "fever"[All Fields]) OR "spotted fever"[All Fields]) AND ("Rickettsia"[MeSH Terms] OR "Rickettsia"[All Fields] OR "rickettsiae"[All Fields] OR "rickettsiales"[MeSH Terms] OR "rickettsiales"[All Fields] OR "rickettsias"[All Fields]) AND ("disease"[MeSH Terms] OR "disease"[All Fields] OR "diseases"[All Fields] OR "disease s"[All Fields] OR "diseased"[All Fields])) OR ("spotted fever group rickettsiosis"[MeSH Terms] OR ("spotted"[All Fields] AND "fever"[All Fields] AND "group"[All Fields] AND "rickettsiosis"[All Fields]) OR "spotted fever group rickettsiosis"[All Fields] OR "rickettsialpox"[All Fields]) OR ("spotted fever group rickettsiosis"[MeSH Terms] OR ("spotted"[All Fields] AND "fever"[All Fields] AND "group"[All Fields] AND "rickettsiosis"[All Fields]) OR "spotted fever group rickettsiosis"[All Fields] OR ("far"[All Fields] AND "eastern"[All Fields] AND "spotted"[All Fields] AND "fever"[All Fields]) OR "far eastern spotted fever"[All Fields]) OR ("spotted fever group rickettsiosis"[MeSH Terms] OR ("spotted"[All Fields] AND "fever"[All Fields] AND "group"[All Fields] AND "rickettsiosis"[All Fields]) OR "spotted fever group rickettsiosis"[All Fields] OR ("north"[All Fields] AND "asian"[All Fields] AND "tick"[All Fields] AND "typhus"[All Fields]) OR "north asian tick typhus"[All Fields]) OR ("spotted fever group rickettsiosis"[MeSH Terms] OR ("spotted"[All Fields] AND "fever"[All Fields] AND "group"[All Fields] AND "rickettsiosis"[All Fields]) OR "spotted fever group rickettsiosis"[All Fields] OR ("queensland"[All Fields] AND "tick"[All Fields] AND "typhus"[All Fields]) OR "queensland tick typhus"[All Fields]) OR ("boutonneuse fever"[MeSH Terms] OR ("boutonneuse"[All Fields] AND "fever"[All Fields]) OR "boutonneuse fever"[All Fields]) OR ("rocky mountain spotted fever"[MeSH Terms] OR ("rocky"[All Fields] AND "mountain"[All Fields] AND "spotted"[All Fields] AND "fever"[All Fields]) OR "rocky mountain spotted fever"[All Fields]) OR (("coxiella"[MeSH Terms] OR "coxiella"[All Fields] OR "coxiellae"[All Fields]) AND "burnetiid"[All Fields]) OR (("coxiella"[MeSH Terms] OR "coxiella"[All Fields]) AND "burneti"[All Fields]) OR (("coxiella"[MeSH Terms] OR "coxiella"[All Fields] OR "coxiellae"[All Fields]) AND "burneti"[All Fields]) OR (("coxiella"[MeSH Terms] OR "coxiella"[All Fields] OR "coxiellae"[All Fields]) AND "burnetti"[All Fields]) OR (("coxiella"[MeSH Terms] OR "coxiella"[All Fields] OR "coxiellae"[All Fields]) AND "burnettii"[All Fields]) OR ("coxiella"[MeSH Terms] OR "coxiella"[All Fields] OR "coxiellae"[All Fields]) OR ("coxsiella"[All Fields] AND "burnetii"[All Fields])) 21,782  5 "Prevalence"[MeSH Terms] 337,863  6 "Prevalence"[MeSH Terms] OR ("Prevalence"[Title/Abstract] OR "Prevalences"[Title/Abstract] OR "period prevalences"[Title/Abstract] OR "point prevalences"[Title/Abstract] OR "Epidemiology"[Title/Abstract] OR "social epidemiologies"[Title/Abstract] OR "controlled before after studies"[Title/Abstract] OR ("controlled before"[Title/Abstract] AND "after studies"[Title/Abstract]) OR ("controlled before"[Title/Abstract] AND "after study"[Title/Abstract]) OR "controlled before after studies"[Title/Abstract] OR "effect modifier epidemiologic"[Title/Abstract] OR ((("effect"[All Fields] OR "effecting"[All Fields] OR "effective"[All Fields] OR "effectively"[All Fields] OR "effectiveness"[All Fields] OR "effectivenesses"[All Fields] OR "effectives"[All Fields] OR "effectivities"[All Fields] OR "effectivity"[All Fields] OR "effects"[All Fields]) AND ("modifiable"[All Fields] OR "modified"[All Fields] OR "modifier"[All Fields] OR "modifiers"[All Fields] OR "modifies"[All Fields] OR "modify"[All Fields] OR "modifying"[All Fields])) AND "psychology"[Title/Abstract]) OR "epidemiologic confounding factors"[Title/Abstract] OR "epidemiologic effect modifier"[Title/Abstract] OR "epidemiologic factors"[Title/Abstract] OR "epidemiologic methods"[Title/Abstract] OR "epidemiologic research"[Title/Abstract] OR "epidemiologic research design"[Title/Abstract] OR "epidemiologic studies"[Title/Abstract] OR "epidemiologic study characteristics"[Title/Abstract] OR "epidemiologic study characteristics as topic"[Title/Abstract] OR "epidemiologic survey"[Title/Abstract] OR "epidemiological research"[Title/Abstract] OR "epidemiometry"[Title/Abstract] OR "historically controlled study"[Title/Abstract] OR "interrupted time series analysis"[Title/Abstract] OR "precipitating factors"[Title/Abstract] OR "sampling studies"[Title/Abstract] OR "epidemiological studies"[Title/Abstract] OR "epidemiologic studies"[Title/Abstract] OR "epidemiological study"[Title/Abstract] OR "epidemiologic study"[Title/Abstract] OR "disease transmission infectious"[Title/Abstract] OR "pathogen transmission"[Title/Abstract] OR "infectious disease transmission"[Title/Abstract] OR "communicable disease transmission"[Title/Abstract] OR "close contact transmission"[Title/Abstract] OR ("Close-Contact"[All Fields] AND "Transmissions"[Title/Abstract]) OR ("Close-Contact"[All Fields] AND "infectious disease transmission"[Title/Abstract]) OR ((("close"[All Fields] OR "closed"[All Fields] OR "closely"[All Fields] OR "closeness"[All Fields] OR "closes"[All Fields] OR "closing"[All Fields] OR "closings"[All Fields]) AND ("contact"[All Fields] OR "contactable"[All Fields] OR "contacted"[All Fields] OR "contacting"[All Fields] OR "contacts"[All Fields])) AND "infectious disease transmission"[Title/Abstract]) OR (("Horizontal"[All Fields] OR "horizontally"[All Fields] OR "horizontals"[All Fields]) AND "transmission of infectious disease"[Title/Abstract]) OR "pathogen transmission horizontal"[Title/Abstract] OR "horizontal transmission of infection"[Title/Abstract] OR "person to person transmission"[Title/Abstract] OR "person to person transmission"[Title/Abstract] OR "droplet transmission of infectious disease"[Title/Abstract] OR (("communicable diseases"[MeSH Terms] OR ("Communicable"[All Fields] AND "diseases"[All Fields]) OR "communicable diseases"[All Fields] OR ("Infectious"[All Fields] AND "Disease"[All Fields]) OR "infectious disease"[All Fields]) AND "droplet transmission"[Title/Abstract]) OR "autochthonous transmission"[Title/Abstract] OR "autochthonous transmissions"[Title/Abstract] OR (("Disease"[MeSH Terms] OR "Disease"[All Fields] OR "diseases"[All Fields] OR "disease s"[All Fields] OR "diseased"[All Fields]) AND "superspreader event"[Title/Abstract]) OR (("Disease"[MeSH Terms] OR "Disease"[All Fields] OR "diseases"[All Fields] OR "disease s"[All Fields] OR "diseased"[All Fields]) AND "superspreader events"[Title/Abstract]) OR "disease superspreading"[Title/Abstract] OR "community transmission"[Title/Abstract] OR "community transmissions"[Title/Abstract] OR "community spread"[Title/Abstract] OR "Incidences"[Title/Abstract] OR "Incidence"[Title/Abstract] OR "secondary attack rate"[Title/Abstract] OR "secondary attack rates"[Title/Abstract] OR "attack rate"[Title/Abstract] OR "attack rates"[Title/Abstract] OR "cumulative incidences"[Title/Abstract] OR "person time rate"[Title/Abstract] OR "person time rate"[Title/Abstract] OR "person time rates"[Title/Abstract]) 1,921,828  7 "China"[MeSH Terms] 261,802  8 "China"[MeSH Terms] OR "China"[Title/Abstract] OR "Sinkiang"[Title/Abstract] OR "inner mongolia"[Title/Abstract] OR "Manchuria"[Title/Abstract] OR "Beijing"[Title/Abstract] OR "hong kong"[Title/Abstract] OR "Macau"[Title/Abstract] OR "Tibet"[Title/Abstract] OR "chinese people s republic"[Title/Abstract] 399,630  9 ("Ticks"[MeSH Terms] OR ("tick s"[All Fields] OR "Ticks"[MeSH Terms] OR "Ticks"[All Fields] OR ("Ticks"[MeSH Terms] OR "Ticks"[All Fields] OR "tick"[All Fields]) OR ("ixodidae"[MeSH Terms] OR "ixodidae"[All Fields] OR "Ticks"[MeSH Terms] OR "Ticks"[All Fields] OR "ixodida"[All Fields]) OR ("Ticks"[MeSH Terms] OR "Ticks"[All Fields]) OR ("ixodidae"[MeSH Terms] OR "ixodidae"[All Fields] OR "Ticks"[MeSH Terms] OR "Ticks"[All Fields] OR "ixodida"[All Fields]) OR ("argasidae"[MeSH Terms] OR "argasidae"[All Fields]) OR ("argas"[MeSH Terms] OR "argas"[All Fields]) OR ("ornithodoros"[MeSH Terms] OR "ornithodoros"[All Fields]) OR ("ixodidae"[MeSH Terms] OR "ixodidae"[All Fields] OR "Ticks"[MeSH Terms] OR "Ticks"[All Fields] OR "ixodida"[All Fields]) OR ("amblyomma"[MeSH Terms] OR "amblyomma"[All Fields]) OR ("dermacentor"[MeSH Terms] OR "dermacentor"[All Fields]) OR ("ixodes"[MeSH Terms] OR "ixodes"[All Fields] OR "ixode"[All Fields] OR "ixodic"[All Fields]) OR ("rhipicephalus"[MeSH Terms] OR "rhipicephalus"[All Fields]) OR "Ixodoidea"[All Fields] OR ("vector borne diseases"[MeSH Terms] OR ("vector"[All Fields] AND "borne"[All Fields] AND "diseases"[All Fields]) OR "vector borne diseases"[All Fields]) OR ("vector borne diseases"[MeSH Terms] OR ("vector"[All Fields] AND "borne"[All Fields] AND "diseases"[All Fields]) OR "vector borne diseases"[All Fields]) OR ("vector borne diseases"[MeSH Terms] OR ("vector"[All Fields] AND "borne"[All Fields] AND "diseases"[All Fields]) OR "vector borne diseases"[All Fields] OR ("vector"[All Fields] AND "borne"[All Fields] AND "disease"[All Fields]) OR "vector borne disease"[All Fields]) OR ("vector borne diseases"[MeSH Terms] OR ("vector"[All Fields] AND "borne"[All Fields] AND "diseases"[All Fields]) OR "vector borne diseases"[All Fields] OR ("vectorborne"[All Fields] AND "diseases"[All Fields]) OR "vectorborne diseases"[All Fields]) OR ("vector borne diseases"[MeSH Terms] OR ("vector"[All Fields] AND "borne"[All Fields] AND "diseases"[All Fields]) OR "vector borne diseases"[All Fields] OR ("vectorborne"[All Fields] AND "disease"[All Fields]) OR "vectorborne disease"[All Fields]) OR ("vector borne diseases"[MeSH Terms] OR ("vector"[All Fields] AND "borne"[All Fields] AND "diseases"[All Fields]) OR "vector borne diseases"[All Fields] OR ("mosquito"[All Fields] AND "borne"[All Fields] AND "diseases"[All Fields]) OR "mosquito borne diseases"[All Fields]) OR ("vector borne diseases"[MeSH Terms] OR ("vector"[All Fields] AND "borne"[All Fields] AND "diseases"[All Fields]) OR "vector borne diseases"[All Fields] OR ("mosquito"[All Fields] AND "borne"[All Fields] AND "disease"[All Fields]) OR "mosquito borne disease"[All Fields]) OR ("vector borne diseases"[MeSH Terms] OR ("vector"[All Fields] AND "borne"[All Fields] AND "diseases"[All Fields]) OR "vector borne diseases"[All Fields] OR ("mosquito"[All Fields] AND "borne"[All Fields] AND "diseases"[All Fields]) OR "mosquito borne diseases"[All Fields]) OR ("vector borne diseases"[MeSH Terms] OR ("vector"[All Fields] AND "borne"[All Fields] AND "diseases"[All Fields]) OR "vector borne diseases"[All Fields] OR ("mosquito"[All Fields] AND "borne"[All Fields] AND "disease"[All Fields]) OR "mosquito borne disease"[All Fields]) OR ("tick borne diseases"[MeSH Terms] OR ("tick borne"[All Fields] AND "diseases"[All Fields]) OR "tick borne diseases"[All Fields] OR ("tick"[All Fields] AND "borne"[All Fields] AND "diseases"[All Fields]) OR "tick borne diseases"[All Fields]) OR ("african swine fever"[MeSH Terms] OR ("african"[All Fields] AND "swine"[All Fields] AND "fever"[All Fields]) OR "african swine fever"[All Fields]) OR ("anaplasmosis"[MeSH Terms] OR "anaplasmosis"[All Fields] OR "anaplasmoses"[All Fields]) OR ("babesiosis"[MeSH Terms] OR "babesiosis"[All Fields] OR "babesioses"[All Fields]) OR ("colorado tick fever"[MeSH Terms] OR ("colorado"[All Fields] AND "tick"[All Fields] AND "fever"[All Fields]) OR "colorado tick fever"[All Fields]) OR ("ehrlichiosis"[MeSH Terms] OR "ehrlichiosis"[All Fields] OR "ehrlichioses"[All Fields]) OR ("encephalitis, tick borne"[MeSH Terms] OR ("encephalitis"[All Fields] AND "tick borne"[All Fields]) OR "tick-borne encephalitis"[All Fields] OR ("encephalitis"[All Fields] AND "tick"[All Fields] AND "borne"[All Fields]) OR "encephalitis tick borne"[All Fields]) OR ("hemorrhagic fever, crimean"[MeSH Terms] OR ("hemorrhagic"[All Fields] AND "fever"[All Fields] AND "crimean"[All Fields]) OR "crimean hemorrhagic fever"[All Fields] OR ("hemorrhagic"[All Fields] AND "fever"[All Fields] AND "crimean"[All Fields]) OR "hemorrhagic fever crimean"[All Fields]) OR ("hemorrhagic fever, omsk"[MeSH Terms] OR ("hemorrhagic"[All Fields] AND "fever"[All Fields] AND "omsk"[All Fields]) OR "omsk hemorrhagic fever"[All Fields] OR ("hemorrhagic"[All Fields] AND "fever"[All Fields] AND "omsk"[All Fields]) OR "hemorrhagic fever omsk"[All Fields]) OR ("kyasanur forest disease"[MeSH Terms] OR ("kyasanur"[All Fields] AND "forest"[All Fields] AND "disease"[All Fields]) OR "kyasanur forest disease"[All Fields]) OR ("lyme disease"[MeSH Terms] OR ("lyme"[All Fields] AND "disease"[All Fields]) OR "lyme disease"[All Fields]) OR ("nairobi sheep disease"[MeSH Terms] OR ("nairobi"[All Fields] AND "sheep"[All Fields] AND "disease"[All Fields]) OR "nairobi sheep disease"[All Fields]) OR ("relapsing fever"[MeSH Terms] OR ("relapsing"[All Fields] AND "fever"[All Fields]) OR "relapsing fever"[All Fields]) OR ("severe fever with thrombocytopenia syndrome"[MeSH Terms] OR ("severe"[All Fields] AND "fever"[All Fields] AND "thrombocytopenia"[All Fields] AND "syndrome"[All Fields]) OR "severe fever with thrombocytopenia syndrome"[All Fields]) OR ("spotted fever group rickettsiosis"[MeSH Terms] OR ("spotted"[All Fields] AND "fever"[All Fields] AND "group"[All Fields] AND "rickettsiosis"[All Fields]) OR "spotted fever group rickettsiosis"[All Fields]) OR ("theileriasis"[MeSH Terms] OR "theileriasis"[All Fields]) OR ("tularaemia"[All Fields] OR "tularemia"[MeSH Terms] OR "tularemia"[All Fields]) OR (("bacterial"[All Fields] OR "bacterially"[All Fields] OR "bacterials"[All Fields]) AND ("genetic vectors"[MeSH Terms] OR ("genetic"[All Fields] AND "vectors"[All Fields]) OR "genetic vectors"[All Fields] OR "vector"[All Fields] OR "vectors"[All Fields] OR "vector s"[All Fields] OR "vectored"[All Fields] OR "vectoring"[All Fields] OR "vectorization"[All Fields] OR "vectorize"[All Fields] OR "vectorized"[All Fields] OR "vectorizing"[All Fields])) OR (("bacterial"[All Fields] OR "bacterially"[All Fields] OR "bacterials"[All Fields]) AND ("genetic vectors"[MeSH Terms] OR ("genetic"[All Fields] AND "vectors"[All Fields]) OR "genetic vectors"[All Fields] OR "vector"[All Fields] OR "vectors"[All Fields] OR "vector s"[All Fields] OR "vectored"[All Fields] OR "vectoring"[All Fields] OR "vectorization"[All Fields] OR "vectorize"[All Fields] OR "vectorized"[All Fields] OR "vectorizing"[All Fields])) OR ("arachnid vectors"[MeSH Terms] OR ("arachnid"[All Fields] AND "vectors"[All Fields]) OR "arachnid vectors"[All Fields]) OR ("arachnid vectors"[MeSH Terms] OR ("arachnid"[All Fields] AND "vectors"[All Fields]) OR "arachnid vectors"[All Fields] OR ("arachnid"[All Fields] AND "vector"[All Fields]) OR "arachnid vector"[All Fields]) OR ("arachnid vectors"[MeSH Terms] OR ("arachnid"[All Fields] AND "vectors"[All Fields]) OR "arachnid vectors"[All Fields] OR ("vectors"[All Fields] AND "arachnid"[All Fields])))) AND ("Rickettsia"[MeSH Terms] OR ("rickettsia infections"[MeSH Terms] OR ("Rickettsia"[All Fields] AND "infections"[All Fields]) OR "rickettsia infections"[All Fields] OR ("boutonneuse fever"[MeSH Terms] OR ("boutonneuse"[All Fields] AND "fever"[All Fields]) OR "boutonneuse fever"[All Fields]) OR ("rocky mountain spotted fever"[MeSH Terms] OR ("rocky"[All Fields] AND "mountain"[All Fields] AND "spotted"[All Fields] AND "fever"[All Fields]) OR "rocky mountain spotted fever"[All Fields]) OR ("typhus, endemic flea borne"[MeSH Terms] OR ("typhus"[All Fields] AND "endemic"[All Fields] AND "flea borne"[All Fields]) OR "endemic flea-borne typhus"[All Fields] OR ("typhus"[All Fields] AND "endemic"[All Fields] AND "flea"[All Fields] AND "borne"[All Fields]) OR "typhus endemic flea borne"[All Fields]) OR ("typhus, epidemic louse borne"[MeSH Terms] OR ("typhus"[All Fields] AND "epidemic"[All Fields] AND "louse borne"[All Fields]) OR "epidemic louse-borne typhus"[All Fields] OR ("typhus"[All Fields] AND "epidemic"[All Fields] AND "louse"[All Fields] AND "borne"[All Fields]) OR "typhus epidemic louse borne"[All Fields]) OR ("rickettsia infections"[MeSH Terms] OR ("Rickettsia"[All Fields] AND "infections"[All Fields]) OR "rickettsia infections"[All Fields] OR ("rickettsial"[All Fields] AND "diseases"[All Fields]) OR "rickettsial diseases"[All Fields]) OR ("rickettsia infections"[MeSH Terms] OR ("Rickettsia"[All Fields] AND "infections"[All Fields]) OR "rickettsia infections"[All Fields] OR ("rickettsial"[All Fields] AND "disease"[All Fields]) OR "rickettsial disease"[All Fields]) OR ("rickettsia infections"[MeSH Terms] OR ("Rickettsia"[All Fields] AND "infections"[All Fields]) OR "rickettsia infections"[All Fields] OR "rickettsiosis"[All Fields]) OR ("rickettsia infections"[MeSH Terms] OR ("Rickettsia"[All Fields] AND "infections"[All Fields]) OR "rickettsia infections"[All Fields] OR "rickettsioses"[All Fields]) OR (("Rickettsia"[MeSH Terms] OR "Rickettsia"[All Fields] OR "rickettsiae"[All Fields] OR "rickettsiales"[MeSH Terms] OR "rickettsiales"[All Fields] OR "rickettsias"[All Fields]) AND ("infectious"[All Fields] OR "infectiousness"[All Fields])) OR ("rickettsia infections"[MeSH Terms] OR ("Rickettsia"[All Fields] AND "infections"[All Fields]) OR "rickettsia infections"[All Fields] OR ("Rickettsia"[All Fields] AND "infection"[All Fields]) OR "rickettsia infection"[All Fields]) OR ("spotted fever group rickettsiosis"[MeSH Terms] OR ("spotted"[All Fields] AND "fever"[All Fields] AND "group"[All Fields] AND "rickettsiosis"[All Fields]) OR "spotted fever group rickettsiosis"[All Fields]) OR ("spotted fever group rickettsiosis"[MeSH Terms] OR ("spotted"[All Fields] AND "fever"[All Fields] AND "group"[All Fields] AND "rickettsiosis"[All Fields]) OR "spotted fever group rickettsiosis"[All Fields] OR ("spotted"[All Fields] AND "fevers"[All Fields]) OR "spotted fevers"[All Fields]) OR ("spotted fever group rickettsiosis"[MeSH Terms] OR ("spotted"[All Fields] AND "fever"[All Fields] AND "group"[All Fields] AND "rickettsiosis"[All Fields]) OR "spotted fever group rickettsiosis"[All Fields] OR ("spotted"[All Fields] AND "fever"[All Fields]) OR "spotted fever"[All Fields]) OR ("spotted fever group rickettsiosis"[MeSH Terms] OR ("spotted"[All Fields] AND "fever"[All Fields] AND "group"[All Fields] AND "rickettsiosis"[All Fields]) OR "spotted fever group rickettsiosis"[All Fields] OR ("tick"[All Fields] AND "borne"[All Fields] AND "lymphadenopathy"[All Fields]) OR "tick borne lymphadenopathy"[All Fields]) OR ("spotted fever group rickettsiosis"[MeSH Terms] OR ("spotted"[All Fields] AND "fever"[All Fields] AND "group"[All Fields] AND "rickettsiosis"[All Fields]) OR "spotted fever group rickettsiosis"[All Fields] OR ("tick"[All Fields] AND "borne"[All Fields] AND "lymphadenopathy"[All Fields]) OR "tick borne lymphadenopathy"[All Fields]) OR ("spotted fever group rickettsiosis"[MeSH Terms] OR ("spotted"[All Fields] AND "fever"[All Fields] AND "group"[All Fields] AND "rickettsiosis"[All Fields]) OR "spotted fever group rickettsiosis"[All Fields] OR ("tick"[All Fields] AND "borne"[All Fields] AND "lymphadenopathies"[All Fields])) OR ("spotted fever group rickettsiosis"[MeSH Terms] OR ("spotted"[All Fields] AND "fever"[All Fields] AND "group"[All Fields] AND "rickettsiosis"[All Fields]) OR "spotted fever group rickettsiosis"[All Fields] OR ("Rickettsia"[All Fields] AND "slovaca"[All Fields] AND "infections"[All Fields]) OR "rickettsia slovaca infections"[All Fields]) OR ("spotted fever group rickettsiosis"[MeSH Terms] OR ("spotted"[All Fields] AND "fever"[All Fields] AND "group"[All Fields] AND "rickettsiosis"[All Fields]) OR "spotted fever group rickettsiosis"[All Fields] OR "tibola"[All Fields]) OR ("spotted fever group rickettsiosis"[MeSH Terms] OR ("spotted"[All Fields] AND "fever"[All Fields] AND "group"[All Fields] AND "rickettsiosis"[All Fields]) OR "spotted fever group rickettsiosis"[All Fields] OR ("african"[All Fields] AND "tick"[All Fields] AND "bite"[All Fields] AND "fever"[All Fields]) OR "african tick bite fever"[All Fields]) OR ("spotted fever group rickettsiosis"[MeSH Terms] OR ("spotted"[All Fields] AND "fever"[All Fields] AND "group"[All Fields] AND "rickettsiosis"[All Fields]) OR "spotted fever group rickettsiosis"[All Fields] OR ("african"[All Fields] AND "tick"[All Fields] AND "bite"[All Fields] AND "fever"[All Fields]) OR "african tick bite fever"[All Fields]) OR ("spotted fever group rickettsiosis"[MeSH Terms] OR ("spotted"[All Fields] AND "fever"[All Fields] AND "group"[All Fields] AND "rickettsiosis"[All Fields]) OR "spotted fever group rickettsiosis"[All Fields] OR ("flinders"[All Fields] AND "island"[All Fields] AND "spotted"[All Fields] AND "fever"[All Fields]) OR "flinders island spotted fever"[All Fields]) OR (("spotted fever group rickettsiosis"[MeSH Terms] OR ("spotted"[All Fields] AND "fever"[All Fields] AND "group"[All Fields] AND "rickettsiosis"[All Fields]) OR "spotted fever group rickettsiosis"[All Fields] OR ("spotted"[All Fields] AND "fever"[All Fields]) OR "spotted fever"[All Fields]) AND ("Rickettsia"[MeSH Terms] OR "Rickettsia"[All Fields] OR "rickettsiae"[All Fields] OR "rickettsiales"[MeSH Terms] OR "rickettsiales"[All Fields] OR "rickettsias"[All Fields]) AND ("disease"[MeSH Terms] OR "disease"[All Fields] OR "diseases"[All Fields] OR "disease s"[All Fields] OR "diseased"[All Fields])) OR ("spotted fever group rickettsiosis"[MeSH Terms] OR ("spotted"[All Fields] AND "fever"[All Fields] AND "group"[All Fields] AND "rickettsiosis"[All Fields]) OR "spotted fever group rickettsiosis"[All Fields] OR "rickettsialpox"[All Fields]) OR ("spotted fever group rickettsiosis"[MeSH Terms] OR ("spotted"[All Fields] AND "fever"[All Fields] AND "group"[All Fields] AND "rickettsiosis"[All Fields]) OR "spotted fever group rickettsiosis"[All Fields] OR ("far"[All Fields] AND "eastern"[All Fields] AND "spotted"[All Fields] AND "fever"[All Fields]) OR "far eastern spotted fever"[All Fields]) OR ("spotted fever group rickettsiosis"[MeSH Terms] OR ("spotted"[All Fields] AND "fever"[All Fields] AND "group"[All Fields] AND "rickettsiosis"[All Fields]) OR "spotted fever group rickettsiosis"[All Fields] OR ("north"[All Fields] AND "asian"[All Fields] AND "tick"[All Fields] AND "typhus"[All Fields]) OR "north asian tick typhus"[All Fields]) OR ("spotted fever group rickettsiosis"[MeSH Terms] OR ("spotted"[All Fields] AND "fever"[All Fields] AND "group"[All Fields] AND "rickettsiosis"[All Fields]) OR "spotted fever group rickettsiosis"[All Fields] OR ("queensland"[All Fields] AND "tick"[All Fields] AND "typhus"[All Fields]) OR "queensland tick typhus"[All Fields]) OR ("boutonneuse fever"[MeSH Terms] OR ("boutonneuse"[All Fields] AND "fever"[All Fields]) OR "boutonneuse fever"[All Fields]) OR ("rocky mountain spotted fever"[MeSH Terms] OR ("rocky"[All Fields] AND "mountain"[All Fields] AND "spotted"[All Fields] AND "fever"[All Fields]) OR "rocky mountain spotted fever"[All Fields]) OR (("coxiella"[MeSH Terms] OR "coxiella"[All Fields] OR "coxiellae"[All Fields]) AND "burnetiid"[All Fields]) OR (("coxiella"[MeSH Terms] OR "coxiella"[All Fields]) AND "burneti"[All Fields]) OR (("coxiella"[MeSH Terms] OR "coxiella"[All Fields] OR "coxiellae"[All Fields]) AND "burneti"[All Fields]) OR (("coxiella"[MeSH Terms] OR "coxiella"[All Fields] OR "coxiellae"[All Fields]) AND "burnetti"[All Fields]) OR (("coxiella"[MeSH Terms] OR "coxiella"[All Fields] OR "coxiellae"[All Fields]) AND "burnettii"[All Fields]) OR ("coxiella"[MeSH Terms] OR "coxiella"[All Fields] OR "coxiellae"[All Fields]) OR ("coxsiella"[All Fields] AND "burnetii"[All Fields]))) 13,706  10 ("Ticks"[MeSH Terms] OR ("tick s"[All Fields] OR "Ticks"[MeSH Terms] OR "Ticks"[All Fields] OR ("Ticks"[MeSH Terms] OR "Ticks"[All Fields] OR "tick"[All Fields]) OR ("ixodidae"[MeSH Terms] OR "ixodidae"[All Fields] OR "Ticks"[MeSH Terms] OR "Ticks"[All Fields] OR "ixodida"[All Fields]) OR ("Ticks"[MeSH Terms] OR "Ticks"[All Fields]) OR ("ixodidae"[MeSH Terms] OR "ixodidae"[All Fields] OR "Ticks"[MeSH Terms] OR "Ticks"[All Fields] OR "ixodida"[All Fields]) OR ("argasidae"[MeSH Terms] OR "argasidae"[All Fields]) OR ("argas"[MeSH Terms] OR "argas"[All Fields]) OR ("ornithodoros"[MeSH Terms] OR "ornithodoros"[All Fields]) OR ("ixodidae"[MeSH Terms] OR "ixodidae"[All Fields] OR "Ticks"[MeSH Terms] OR "Ticks"[All Fields] OR "ixodida"[All Fields]) OR ("amblyomma"[MeSH Terms] OR "amblyomma"[All Fields]) OR ("dermacentor"[MeSH Terms] OR "dermacentor"[All Fields]) OR ("ixodes"[MeSH Terms] OR "ixodes"[All Fields] OR "ixode"[All Fields] OR "ixodic"[All Fields]) OR ("rhipicephalus"[MeSH Terms] OR "rhipicephalus"[All Fields]) OR "Ixodoidea"[All Fields] OR ("vector borne diseases"[MeSH Terms] OR ("vector"[All Fields] AND "borne"[All Fields] AND "diseases"[All Fields]) OR "vector borne diseases"[All Fields]) OR ("vector borne diseases"[MeSH Terms] OR ("vector"[All Fields] AND "borne"[All Fields] AND "diseases"[All Fields]) OR "vector borne diseases"[All Fields]) OR ("vector borne diseases"[MeSH Terms] OR ("vector"[All Fields] AND "borne"[All Fields] AND "diseases"[All Fields]) OR "vector borne diseases"[All Fields] OR ("vector"[All Fields] AND "borne"[All Fields] AND "Disease"[All Fields]) OR "vector borne disease"[All Fields]) OR ("vector borne diseases"[MeSH Terms] OR ("vector"[All Fields] AND "borne"[All Fields] AND "diseases"[All Fields]) OR "vector borne diseases"[All Fields] OR ("vectorborne"[All Fields] AND "diseases"[All Fields]) OR "vectorborne diseases"[All Fields]) OR ("vector borne diseases"[MeSH Terms] OR ("vector"[All Fields] AND "borne"[All Fields] AND "diseases"[All Fields]) OR "vector borne diseases"[All Fields] OR ("vectorborne"[All Fields] AND "Disease"[All Fields]) OR "vectorborne disease"[All Fields]) OR ("vector borne diseases"[MeSH Terms] OR ("vector"[All Fields] AND "borne"[All Fields] AND "diseases"[All Fields]) OR "vector borne diseases"[All Fields] OR ("mosquito"[All Fields] AND "borne"[All Fields] AND "diseases"[All Fields]) OR "mosquito borne diseases"[All Fields]) OR ("vector borne diseases"[MeSH Terms] OR ("vector"[All Fields] AND "borne"[All Fields] AND "diseases"[All Fields]) OR "vector borne diseases"[All Fields] OR ("mosquito"[All Fields] AND "borne"[All Fields] AND "Disease"[All Fields]) OR "mosquito borne disease"[All Fields]) OR ("vector borne diseases"[MeSH Terms] OR ("vector"[All Fields] AND "borne"[All Fields] AND "diseases"[All Fields]) OR "vector borne diseases"[All Fields] OR ("mosquito"[All Fields] AND "borne"[All Fields] AND "diseases"[All Fields]) OR "mosquito borne diseases"[All Fields]) OR ("vector borne diseases"[MeSH Terms] OR ("vector"[All Fields] AND "borne"[All Fields] AND "diseases"[All Fields]) OR "vector borne diseases"[All Fields] OR ("mosquito"[All Fields] AND "borne"[All Fields] AND "Disease"[All Fields]) OR "mosquito borne disease"[All Fields]) OR ("tick borne diseases"[MeSH Terms] OR ("tick borne"[All Fields] AND "diseases"[All Fields]) OR "tick borne diseases"[All Fields] OR ("tick"[All Fields] AND "borne"[All Fields] AND "diseases"[All Fields]) OR "tick borne diseases"[All Fields]) OR ("african swine fever"[MeSH Terms] OR ("african"[All Fields] AND "swine"[All Fields] AND "fever"[All Fields]) OR "african swine fever"[All Fields]) OR ("anaplasmosis"[MeSH Terms] OR "anaplasmosis"[All Fields] OR "anaplasmoses"[All Fields]) OR ("babesiosis"[MeSH Terms] OR "babesiosis"[All Fields] OR "babesioses"[All Fields]) OR ("colorado tick fever"[MeSH Terms] OR ("colorado"[All Fields] AND "tick"[All Fields] AND "fever"[All Fields]) OR "colorado tick fever"[All Fields]) OR ("ehrlichiosis"[MeSH Terms] OR "ehrlichiosis"[All Fields] OR "ehrlichioses"[All Fields]) OR ("encephalitis, tick borne"[MeSH Terms] OR ("encephalitis"[All Fields] AND "tick borne"[All Fields]) OR "tick-borne encephalitis"[All Fields] OR ("encephalitis"[All Fields] AND "tick"[All Fields] AND "borne"[All Fields]) OR "encephalitis tick borne"[All Fields]) OR ("hemorrhagic fever, crimean"[MeSH Terms] OR ("hemorrhagic"[All Fields] AND "fever"[All Fields] AND "crimean"[All Fields]) OR "crimean hemorrhagic fever"[All Fields] OR ("hemorrhagic"[All Fields] AND "fever"[All Fields] AND "crimean"[All Fields]) OR "hemorrhagic fever crimean"[All Fields]) OR ("hemorrhagic fever, omsk"[MeSH Terms] OR ("hemorrhagic"[All Fields] AND "fever"[All Fields] AND "omsk"[All Fields]) OR "omsk hemorrhagic fever"[All Fields] OR ("hemorrhagic"[All Fields] AND "fever"[All Fields] AND "omsk"[All Fields]) OR "hemorrhagic fever omsk"[All Fields]) OR ("kyasanur forest disease"[MeSH Terms] OR ("kyasanur"[All Fields] AND "forest"[All Fields] AND "Disease"[All Fields]) OR "kyasanur forest disease"[All Fields]) OR ("lyme disease"[MeSH Terms] OR ("lyme"[All Fields] AND "Disease"[All Fields]) OR "lyme disease"[All Fields]) OR ("nairobi sheep disease"[MeSH Terms] OR ("nairobi"[All Fields] AND "sheep"[All Fields] AND "Disease"[All Fields]) OR "nairobi sheep disease"[All Fields]) OR ("relapsing fever"[MeSH Terms] OR ("relapsing"[All Fields] AND "fever"[All Fields]) OR "relapsing fever"[All Fields]) OR ("severe fever with thrombocytopenia syndrome"[MeSH Terms] OR ("severe"[All Fields] AND "fever"[All Fields] AND "thrombocytopenia"[All Fields] AND "syndrome"[All Fields]) OR "severe fever with thrombocytopenia syndrome"[All Fields]) OR ("spotted fever group rickettsiosis"[MeSH Terms] OR ("spotted"[All Fields] AND "fever"[All Fields] AND "group"[All Fields] AND "rickettsiosis"[All Fields]) OR "spotted fever group rickettsiosis"[All Fields]) OR ("theileriasis"[MeSH Terms] OR "theileriasis"[All Fields]) OR ("tularaemia"[All Fields] OR "tularemia"[MeSH Terms] OR "tularemia"[All Fields]) OR (("bacterial"[All Fields] OR "bacterially"[All Fields] OR "bacterials"[All Fields]) AND ("genetic vectors"[MeSH Terms] OR ("genetic"[All Fields] AND "vectors"[All Fields]) OR "genetic vectors"[All Fields] OR "vector"[All Fields] OR "vectors"[All Fields] OR "vector s"[All Fields] OR "vectored"[All Fields] OR "vectoring"[All Fields] OR "vectorization"[All Fields] OR "vectorize"[All Fields] OR "vectorized"[All Fields] OR "vectorizing"[All Fields])) OR (("bacterial"[All Fields] OR "bacterially"[All Fields] OR "bacterials"[All Fields]) AND ("genetic vectors"[MeSH Terms] OR ("genetic"[All Fields] AND "vectors"[All Fields]) OR "genetic vectors"[All Fields] OR "vector"[All Fields] OR "vectors"[All Fields] OR "vector s"[All Fields] OR "vectored"[All Fields] OR "vectoring"[All Fields] OR "vectorization"[All Fields] OR "vectorize"[All Fields] OR "vectorized"[All Fields] OR "vectorizing"[All Fields])) OR ("arachnid vectors"[MeSH Terms] OR ("arachnid"[All Fields] AND "vectors"[All Fields]) OR "arachnid vectors"[All Fields]) OR ("arachnid vectors"[MeSH Terms] OR ("arachnid"[All Fields] AND "vectors"[All Fields]) OR "arachnid vectors"[All Fields] OR ("arachnid"[All Fields] AND "vector"[All Fields]) OR "arachnid vector"[All Fields]) OR ("arachnid vectors"[MeSH Terms] OR ("arachnid"[All Fields] AND "vectors"[All Fields]) OR "arachnid vectors"[All Fields] OR ("vectors"[All Fields] AND "arachnid"[All Fields])))) AND ("Rickettsia"[MeSH Terms] OR ("rickettsia infections"[MeSH Terms] OR ("Rickettsia"[All Fields] AND "infections"[All Fields]) OR "rickettsia infections"[All Fields] OR ("boutonneuse fever"[MeSH Terms] OR ("boutonneuse"[All Fields] AND "fever"[All Fields]) OR "boutonneuse fever"[All Fields]) OR ("rocky mountain spotted fever"[MeSH Terms] OR ("rocky"[All Fields] AND "mountain"[All Fields] AND "spotted"[All Fields] AND "fever"[All Fields]) OR "rocky mountain spotted fever"[All Fields]) OR ("typhus, endemic flea borne"[MeSH Terms] OR ("typhus"[All Fields] AND "endemic"[All Fields] AND "flea borne"[All Fields]) OR "endemic flea-borne typhus"[All Fields] OR ("typhus"[All Fields] AND "endemic"[All Fields] AND "flea"[All Fields] AND "borne"[All Fields]) OR "typhus endemic flea borne"[All Fields]) OR ("typhus, epidemic louse borne"[MeSH Terms] OR ("typhus"[All Fields] AND "epidemic"[All Fields] AND "louse borne"[All Fields]) OR "epidemic louse-borne typhus"[All Fields] OR ("typhus"[All Fields] AND "epidemic"[All Fields] AND "louse"[All Fields] AND "borne"[All Fields]) OR "typhus epidemic louse borne"[All Fields]) OR ("rickettsia infections"[MeSH Terms] OR ("Rickettsia"[All Fields] AND "infections"[All Fields]) OR "rickettsia infections"[All Fields] OR ("rickettsial"[All Fields] AND "diseases"[All Fields]) OR "rickettsial diseases"[All Fields]) OR ("rickettsia infections"[MeSH Terms] OR ("Rickettsia"[All Fields] AND "infections"[All Fields]) OR "rickettsia infections"[All Fields] OR ("rickettsial"[All Fields] AND "Disease"[All Fields]) OR "rickettsial disease"[All Fields]) OR ("rickettsia infections"[MeSH Terms] OR ("Rickettsia"[All Fields] AND "infections"[All Fields]) OR "rickettsia infections"[All Fields] OR "rickettsiosis"[All Fields]) OR ("rickettsia infections"[MeSH Terms] OR ("Rickettsia"[All Fields] AND "infections"[All Fields]) OR "rickettsia infections"[All Fields] OR "rickettsioses"[All Fields]) OR (("Rickettsia"[MeSH Terms] OR "Rickettsia"[All Fields] OR "rickettsiae"[All Fields] OR "rickettsiales"[MeSH Terms] OR "rickettsiales"[All Fields] OR "rickettsias"[All Fields]) AND ("Infectious"[All Fields] OR "infectiousness"[All Fields])) OR ("rickettsia infections"[MeSH Terms] OR ("Rickettsia"[All Fields] AND "infections"[All Fields]) OR "rickettsia infections"[All Fields] OR ("Rickettsia"[All Fields] AND "Infection"[All Fields]) OR "rickettsia infection"[All Fields]) OR ("spotted fever group rickettsiosis"[MeSH Terms] OR ("spotted"[All Fields] AND "fever"[All Fields] AND "group"[All Fields] AND "rickettsiosis"[All Fields]) OR "spotted fever group rickettsiosis"[All Fields]) OR ("spotted fever group rickettsiosis"[MeSH Terms] OR ("spotted"[All Fields] AND "fever"[All Fields] AND "group"[All Fields] AND "rickettsiosis"[All Fields]) OR "spotted fever group rickettsiosis"[All Fields] OR ("spotted"[All Fields] AND "fevers"[All Fields]) OR "spotted fevers"[All Fields]) OR ("spotted fever group rickettsiosis"[MeSH Terms] OR ("spotted"[All Fields] AND "fever"[All Fields] AND "group"[All Fields] AND "rickettsiosis"[All Fields]) OR "spotted fever group rickettsiosis"[All Fields] OR ("spotted"[All Fields] AND "fever"[All Fields]) OR "spotted fever"[All Fields]) OR ("spotted fever group rickettsiosis"[MeSH Terms] OR ("spotted"[All Fields] AND "fever"[All Fields] AND "group"[All Fields] AND "rickettsiosis"[All Fields]) OR "spotted fever group rickettsiosis"[All Fields] OR ("tick"[All Fields] AND "borne"[All Fields] AND "lymphadenopathy"[All Fields]) OR "tick borne lymphadenopathy"[All Fields]) OR ("spotted fever group rickettsiosis"[MeSH Terms] OR ("spotted"[All Fields] AND "fever"[All Fields] AND "group"[All Fields] AND "rickettsiosis"[All Fields]) OR "spotted fever group rickettsiosis"[All Fields] OR ("tick"[All Fields] AND "borne"[All Fields] AND "lymphadenopathy"[All Fields]) OR "tick borne lymphadenopathy"[All Fields]) OR ("spotted fever group rickettsiosis"[MeSH Terms] OR ("spotted"[All Fields] AND "fever"[All Fields] AND "group"[All Fields] AND "rickettsiosis"[All Fields]) OR "spotted fever group rickettsiosis"[All Fields] OR ("tick"[All Fields] AND "borne"[All Fields] AND "lymphadenopathies"[All Fields])) OR ("spotted fever group rickettsiosis"[MeSH Terms] OR ("spotted"[All Fields] AND "fever"[All Fields] AND "group"[All Fields] AND "rickettsiosis"[All Fields]) OR "spotted fever group rickettsiosis"[All Fields] OR ("Rickettsia"[All Fields] AND "slovaca"[All Fields] AND "infections"[All Fields]) OR "rickettsia slovaca infections"[All Fields]) OR ("spotted fever group rickettsiosis"[MeSH Terms] OR ("spotted"[All Fields] AND "fever"[All Fields] AND "group"[All Fields] AND "rickettsiosis"[All Fields]) OR "spotted fever group rickettsiosis"[All Fields] OR "tibola"[All Fields]) OR ("spotted fever group rickettsiosis"[MeSH Terms] OR ("spotted"[All Fields] AND "fever"[All Fields] AND "group"[All Fields] AND "rickettsiosis"[All Fields]) OR "spotted fever group rickettsiosis"[All Fields] OR ("african"[All Fields] AND "tick"[All Fields] AND "bite"[All Fields] AND "fever"[All Fields]) OR "african tick bite fever"[All Fields]) OR ("spotted fever group rickettsiosis"[MeSH Terms] OR ("spotted"[All Fields] AND "fever"[All Fields] AND "group"[All Fields] AND "rickettsiosis"[All Fields]) OR "spotted fever group rickettsiosis"[All Fields] OR ("african"[All Fields] AND "tick"[All Fields] AND "bite"[All Fields] AND "fever"[All Fields]) OR "african tick bite fever"[All Fields]) OR ("spotted fever group rickettsiosis"[MeSH Terms] OR ("spotted"[All Fields] AND "fever"[All Fields] AND "group"[All Fields] AND "rickettsiosis"[All Fields]) OR "spotted fever group rickettsiosis"[All Fields] OR ("flinders"[All Fields] AND "island"[All Fields] AND "spotted"[All Fields] AND "fever"[All Fields]) OR "flinders island spotted fever"[All Fields]) OR (("spotted fever group rickettsiosis"[MeSH Terms] OR ("spotted"[All Fields] AND "fever"[All Fields] AND "group"[All Fields] AND "rickettsiosis"[All Fields]) OR "spotted fever group rickettsiosis"[All Fields] OR ("spotted"[All Fields] AND "fever"[All Fields]) OR "spotted fever"[All Fields]) AND ("Rickettsia"[MeSH Terms] OR "Rickettsia"[All Fields] OR "rickettsiae"[All Fields] OR "rickettsiales"[MeSH Terms] OR "rickettsiales"[All Fields] OR "rickettsias"[All Fields]) AND ("Disease"[MeSH Terms] OR "Disease"[All Fields] OR "diseases"[All Fields] OR "disease s"[All Fields] OR "diseased"[All Fields])) OR ("spotted fever group rickettsiosis"[MeSH Terms] OR ("spotted"[All Fields] AND "fever"[All Fields] AND "group"[All Fields] AND "rickettsiosis"[All Fields]) OR "spotted fever group rickettsiosis"[All Fields] OR "rickettsialpox"[All Fields]) OR ("spotted fever group rickettsiosis"[MeSH Terms] OR ("spotted"[All Fields] AND "fever"[All Fields] AND "group"[All Fields] AND "rickettsiosis"[All Fields]) OR "spotted fever group rickettsiosis"[All Fields] OR ("far"[All Fields] AND "eastern"[All Fields] AND "spotted"[All Fields] AND "fever"[All Fields]) OR "far eastern spotted fever"[All Fields]) OR ("spotted fever group rickettsiosis"[MeSH Terms] OR ("spotted"[All Fields] AND "fever"[All Fields] AND "group"[All Fields] AND "rickettsiosis"[All Fields]) OR "spotted fever group rickettsiosis"[All Fields] OR ("north"[All Fields] AND "asian"[All Fields] AND "tick"[All Fields] AND "typhus"[All Fields]) OR "north asian tick typhus"[All Fields]) OR ("spotted fever group rickettsiosis"[MeSH Terms] OR ("spotted"[All Fields] AND "fever"[All Fields] AND "group"[All Fields] AND "rickettsiosis"[All Fields]) OR "spotted fever group rickettsiosis"[All Fields] OR ("queensland"[All Fields] AND "tick"[All Fields] AND "typhus"[All Fields]) OR "queensland tick typhus"[All Fields]) OR ("boutonneuse fever"[MeSH Terms] OR ("boutonneuse"[All Fields] AND "fever"[All Fields]) OR "boutonneuse fever"[All Fields]) OR ("rocky mountain spotted fever"[MeSH Terms] OR ("rocky"[All Fields] AND "mountain"[All Fields] AND "spotted"[All Fields] AND "fever"[All Fields]) OR "rocky mountain spotted fever"[All Fields]) OR (("coxiella"[MeSH Terms] OR "coxiella"[All Fields] OR "coxiellae"[All Fields]) AND "burnetiid"[All Fields]) OR (("coxiella"[MeSH Terms] OR "coxiella"[All Fields]) AND "burneti"[All Fields]) OR (("coxiella"[MeSH Terms] OR "coxiella"[All Fields] OR "coxiellae"[All Fields]) AND "burneti"[All Fields]) OR (("coxiella"[MeSH Terms] OR "coxiella"[All Fields] OR "coxiellae"[All Fields]) AND "burnetti"[All Fields]) OR (("coxiella"[MeSH Terms] OR "coxiella"[All Fields] OR "coxiellae"[All Fields]) AND "burnettii"[All Fields]) OR ("coxiella"[MeSH Terms] OR "coxiella"[All Fields] OR "coxiellae"[All Fields]) OR ("coxsiella"[All Fields] AND "burnetii"[All Fields]))) AND ("Prevalence"[MeSH Terms] OR ("Prevalence"[Title/Abstract] OR "Prevalences"[Title/Abstract] OR "period prevalences"[Title/Abstract] OR "point prevalences"[Title/Abstract] OR "Epidemiology"[Title/Abstract] OR "social epidemiologies"[Title/Abstract] OR "controlled before after studies"[Title/Abstract] OR ("controlled before"[Title/Abstract] AND "after studies"[Title/Abstract]) OR ("controlled before"[Title/Abstract] AND "after study"[Title/Abstract]) OR "controlled before after studies"[Title/Abstract] OR "effect modifier epidemiologic"[Title/Abstract] OR ((("effect"[All Fields] OR "effecting"[All Fields] OR "effective"[All Fields] OR "effectively"[All Fields] OR "effectiveness"[All Fields] OR "effectivenesses"[All Fields] OR "effectives"[All Fields] OR "effectivities"[All Fields] OR "effectivity"[All Fields] OR "effects"[All Fields]) AND ("modifiable"[All Fields] OR "modified"[All Fields] OR "modifier"[All Fields] OR "modifiers"[All Fields] OR "modifies"[All Fields] OR "modify"[All Fields] OR "modifying"[All Fields])) AND "psychology"[Title/Abstract]) OR "epidemiologic confounding factors"[Title/Abstract] OR "epidemiologic effect modifier"[Title/Abstract] OR "epidemiologic factors"[Title/Abstract] OR "epidemiologic methods"[Title/Abstract] OR "epidemiologic research"[Title/Abstract] OR "epidemiologic research design"[Title/Abstract] OR "epidemiologic studies"[Title/Abstract] OR "epidemiologic study characteristics"[Title/Abstract] OR "epidemiologic study characteristics as topic"[Title/Abstract] OR "epidemiologic survey"[Title/Abstract] OR "epidemiological research"[Title/Abstract] OR "epidemiometry"[Title/Abstract] OR "historically controlled study"[Title/Abstract] OR "interrupted time series analysis"[Title/Abstract] OR "precipitating factors"[Title/Abstract] OR "sampling studies"[Title/Abstract] OR "epidemiological studies"[Title/Abstract] OR "epidemiologic studies"[Title/Abstract] OR "epidemiological study"[Title/Abstract] OR "epidemiologic study"[Title/Abstract] OR "disease transmission infectious"[Title/Abstract] OR "pathogen transmission"[Title/Abstract] OR "infectious disease transmission"[Title/Abstract] OR "communicable disease transmission"[Title/Abstract] OR "close contact transmission"[Title/Abstract] OR ("Close-Contact"[All Fields] AND "Transmissions"[Title/Abstract]) OR ("Close-Contact"[All Fields] AND "infectious disease transmission"[Title/Abstract]) OR ((("close"[All Fields] OR "closed"[All Fields] OR "closely"[All Fields] OR "closeness"[All Fields] OR "closes"[All Fields] OR "closing"[All Fields] OR "closings"[All Fields]) AND ("contact"[All Fields] OR "contactable"[All Fields] OR "contacted"[All Fields] OR "contacting"[All Fields] OR "contacts"[All Fields])) AND "infectious disease transmission"[Title/Abstract]) OR (("Horizontal"[All Fields] OR "horizontally"[All Fields] OR "horizontals"[All Fields]) AND "transmission of infectious disease"[Title/Abstract]) OR "pathogen transmission horizontal"[Title/Abstract] OR "horizontal transmission of infection"[Title/Abstract] OR "person to person transmission"[Title/Abstract] OR "person to person transmission"[Title/Abstract] OR "droplet transmission of infectious disease"[Title/Abstract] OR (("communicable diseases"[MeSH Terms] OR ("Communicable"[All Fields] AND "diseases"[All Fields]) OR "communicable diseases"[All Fields] OR ("Infectious"[All Fields] AND "Disease"[All Fields]) OR "infectious disease"[All Fields]) AND "droplet transmission"[Title/Abstract]) OR "autochthonous transmission"[Title/Abstract] OR "autochthonous transmissions"[Title/Abstract] OR (("Disease"[MeSH Terms] OR "Disease"[All Fields] OR "diseases"[All Fields] OR "disease s"[All Fields] OR "diseased"[All Fields]) AND "superspreader event"[Title/Abstract]) OR (("Disease"[MeSH Terms] OR "Disease"[All Fields] OR "diseases"[All Fields] OR "disease s"[All Fields] OR "diseased"[All Fields]) AND "superspreader events"[Title/Abstract]) OR "disease superspreading"[Title/Abstract] OR "community transmission"[Title/Abstract] OR "community transmissions"[Title/Abstract] OR "community spread"[Title/Abstract] OR "Incidences"[Title/Abstract] OR "Incidence"[Title/Abstract] OR "secondary attack rate"[Title/Abstract] OR "secondary attack rates"[Title/Abstract] OR "attack rate"[Title/Abstract] OR "attack rates"[Title/Abstract] OR "cumulative incidences"[Title/Abstract] OR "person time rate"[Title/Abstract] OR "person time rate"[Title/Abstract] OR "person time rates"[Title/Abstract])) AND ("China"[MeSH Terms] OR ("China"[Title/Abstract] OR "Sinkiang"[Title/Abstract] OR "inner mongolia"[Title/Abstract] OR "Manchuria"[Title/Abstract] OR "Beijing"[Title/Abstract] OR "hong kong"[Title/Abstract] OR "Macau"[Title/Abstract] OR "Tibet"[Title/Abstract] OR "chinese people s republic"[Title/Abstract])) 121 | 121 |
| Embase | #1 'tick'/exp 27463  #2 ticks:ti,ab,kw OR tick:ti,ab,kw OR ixodida:ti,ab,kw OR ixodidas:ti,ab,kw OR argasidae:ti,ab,kw OR argas:ti,ab,kw OR ornithodoros:ti,ab,kw OR ixodidae:ti,ab,kw OR amblyomma:ti,ab,kw OR dermacentor:ti,ab,kw OR ixodes:ti,ab,kw OR rhipicephalus:ti,ab,kw OR ixodoidea:ti,ab,kw OR 'vector borne disease':ti,ab,kw OR 'vector-borne diseases':ti,ab,kw OR 'vector-borne disease':ti,ab,kw OR 'vectorborne diseases':ti,ab,kw OR 'vectorborne disease':ti,ab,kw OR 'mosquito-borne diseases':ti,ab,kw OR 'mosquito-borne disease':ti,ab,kw OR 'mosquito borne diseases':ti,ab,kw OR 'mosquito borne disease':ti,ab,kw OR 'tick-borne diseases':ti,ab,kw OR 'african swine fever':ti,ab,kw OR anaplasmosis:ti,ab,kw OR babesiosis:ti,ab,kw OR 'colorado tick fever':ti,ab,kw OR ehrlichiosis:ti,ab,kw OR 'encephalitis, tick-borne':ti,ab,kw OR 'hemorrhagic fever, crimean':ti,ab,kw OR 'hemorrhagic fever, omsk':ti,ab,kw OR 'kyasanur forest disease':ti,ab,kw OR 'lyme disease':ti,ab,kw OR 'relapsing fever':ti,ab,kw OR 'nairobi sheep disease':ti,ab,kw OR 'severe fever with thrombocytopenia syndrome':ti,ab,kw OR 'spotted fever group rickettsiosis':ti,ab,kw OR theileriasis:ti,ab,kw OR tularemia:ti,ab,kw OR 'bacterial vector':ti,ab,kw OR 'bacterial vectors':ti,ab,kw OR 'arachnid vector':ti,ab,kw OR 'arachnid vectors':ti,ab,kw 66706  #3 #1 OR #2 68779  #4 'rickettsia'/exp 9104  #5 rickettsia:ti,ab,kw OR 'rickettsia infections':ti,ab,kw OR 'typhus, endemic flea-borne':ti,ab,kw OR 'typhus, epidemic louse-borne':ti,ab,kw OR 'rickettsial diseases':ti,ab,kw OR 'rickettsial disease':ti,ab,kw OR rickettsiosis:ti,ab,kw OR rickettsioses:ti,ab,kw OR 'rickettsia infectious':ti,ab,kw OR 'rickettsia infection':ti,ab,kw OR rhipicephalus:ti,ab,kw OR 'spotted fever group rickettsiosis':ti,ab,kw OR 'spotted fevers':ti,ab,kw OR 'spotted fever':ti,ab,kw OR 'spotted fever rickettsiae disease':ti,ab,kw OR 'tick-borne lymphadenopathy':ti,ab,kw OR 'tick borne lymphadenopathy':ti,ab,kw OR 'tick-borne lymphadenopathies':ti,ab,kw OR 'rickettsia slovaca infections':ti,ab,kw OR tibola:ti,ab,kw OR 'african tick-bite fever':ti,ab,kw OR 'african tick bite fever':ti,ab,kw OR rickettsialpox:ti,ab,kw OR 'flinders island spotted fever':ti,ab,kw OR 'far eastern spotted fever':ti,ab,kw OR 'north asian tick typhus':ti,ab,kw OR 'queensland tick typhus':ti,ab,kw OR 'boutonneuse fever':ti,ab,kw OR 'rocky mountain spotted fever':ti,ab,kw OR 'coxiella burnetiid':ti,ab,kw OR 'burnetia burneti':ti,ab,kw OR 'coxiella burneti':ti,ab,kw OR 'coxiella burnetti':ti,ab,kw OR 'coxiella burnettii':ti,ab,kw OR 'coxiella diaporica':ti,ab,kw OR 'coxsiella burnetii':ti,ab,kw 16631  #6 #4 OR #5 18669  #7 'prevalence'/exp 933872  #8 prevalence:ti,ab,kw OR prevalences:ti,ab,kw OR 'period prevalences':ti,ab,kw OR 'point prevalences':ti,ab,kw OR epidemiology:ti,ab,kw OR 'social epidemiologies':ti,ab,kw OR 'controlled before after studies':ti,ab,kw OR ('controlled before':ti,ab,kw AND 'after studies':ti,ab,kw) OR ('controlled before':ti,ab,kw AND 'after study':ti,ab,kw) OR 'controlled before-after studies':ti,ab,kw OR 'effect modifier, epidemiologic':ti,ab,kw OR ('effect modifiers':ti,ab,kw AND psychology:ti,ab,kw) OR 'epidemiologic confounding factors':ti,ab,kw OR 'epidemiologic effect modifier':ti,ab,kw OR 'epidemiologic factors':ti,ab,kw OR 'epidemiologic methods':ti,ab,kw OR 'epidemiologic research':ti,ab,kw OR 'epidemiologic research design':ti,ab,kw OR 'epidemiologic study characteristics':ti,ab,kw OR 'epidemiologic survey':ti,ab,kw OR 'epidemiological research':ti,ab,kw OR epidemiometry:ti,ab,kw OR 'historically controlled study':ti,ab,kw OR 'interrupted time series analysis':ti,ab,kw OR 'precipitating factors':ti,ab,kw OR 'sampling studies':ti,ab,kw OR 'epidemiological studies':ti,ab,kw OR 'epidemiologic studies':ti,ab,kw OR 'epidemiological study':ti,ab,kw OR 'epidemiologic study':ti,ab,kw OR 'disease transmission, infectious':ti,ab,kw OR 'pathogen transmission':ti,ab,kw OR 'infectious disease transmission':ti,ab,kw OR 'communicable disease transmission':ti,ab,kw OR 'close-contact transmission':ti,ab,kw OR 'close-contact transmissions':ti,ab,kw OR 'close-contact infectious disease transmission':ti,ab,kw OR 'close contact infectious disease transmission':ti,ab,kw OR 'horizontal transmission of infectious disease':ti,ab,kw OR 'pathogen transmission, horizontal':ti,ab,kw OR 'horizontal transmission of infection':ti,ab,kw OR 'person-to-person transmission':ti,ab,kw OR 'person to person transmission':ti,ab,kw OR 'droplet transmission of infectious disease':ti,ab,kw OR 'infectious disease droplet transmission':ti,ab,kw OR 'autochthonous transmission':ti,ab,kw OR 'autochthonous transmissions':ti,ab,kw OR 'disease superspreader event':ti,ab,kw OR 'disease superspreader events':ti,ab,kw OR 'disease superspreading':ti,ab,kw OR 'disease superspreadings':ti,ab,kw OR 'community transmission':ti,ab,kw OR 'community transmissions':ti,ab,kw OR 'community spread':ti,ab,kw OR incidences:ti,ab,kw OR incidence:ti,ab,kw OR 'secondary attack rate':ti,ab,kw OR 'secondary attack rates':ti,ab,kw OR 'attack rate':ti,ab,kw OR 'attack rates':ti,ab,kw OR 'cumulative incidences':ti,ab,kw OR 'person-time rate':ti,ab,kw OR 'person time rate':ti,ab,kw OR 'person-time rates':ti,ab,kw 2601279  #9 #7 OR #8 2805155  #10 'china'/exp 310294  #11 china:ti,ab,kw OR sinkiang:ti,ab,kw OR 'inner mongolia':ti,ab,kw OR manchuria:ti,ab,kw OR beijing:ti,ab,kw OR 'hong kong':ti,ab,kw OR macau:ti,ab,kw OR tibet:ti,ab,kw OR 'chinese peoples republic':ti,ab,kw 356471  #12 #10 OR #11 429812  #13 #3 AND #6 9372  #14 #9 AND #12 AND #13 113 | 113 |
| Cochrane Library | #1 MeSH descriptor: [Ticks] explode all trees 34  #2 (Tick):ti,ab,kw OR (Ticks):ti,ab,kw OR (Tick):ti,ab,kw OR (Ixodida):ti,ab,kw OR (Ixodidas):ti,ab,kw 436  #3 (Ixodidae):ti,ab,kw OR (Argasidae):ti,ab,kw OR (Argas):ti,ab,kw OR (Ornithodoros):ti,ab,kw OR (Ixodidae):ti,ab,kw 10  #4 (Amblyomma):ti,ab,kw OR (Dermacentor):ti,ab,kw OR (Ixodes):ti,ab,kw OR (Rhipicephalus):ti,ab,kw OR (Ixodoidea):ti,ab,kw 66  #5 (Vector Borne Diseases):ti,ab,kw OR (Vector Borne Disease):ti,ab,kw OR (Vector-Borne Diseases):ti,ab,kw OR (Vector-Borne Disease):ti,ab,kw OR (Vectorborne Diseases):ti,ab,kw 111  #6 (Vectorborne Disease):ti,ab,kw OR (Mosquito-Borne Diseases):ti,ab,kw OR (Mosquito-Borne Disease):ti,ab,kw OR (Mosquito Borne Diseases):ti,ab,kw OR (Mosquito Borne Disease):ti,ab,kw 135  #7 (Tick-Borne Diseases):ti,ab,kw OR (African Swine Fever):ti,ab,kw OR (Anaplasmosis):ti,ab,kw OR (Babesiosis):ti,ab,kw OR (Colorado Tick Fever):ti,ab,kw 55  #8 (Ehrlichiosis):ti,ab,kw OR (Encephalitis, Tick-Borne):ti,ab,kw OR (Hemorrhagic Fever, Crimean):ti,ab,kw OR (Hemorrhagic Fever, Omsk):ti,ab,kw OR (Kyasanur Forest Disease):ti,ab,kw 131  #9 (Lyme Disease):ti,ab,kw OR (Nairobi Sheep Disease):ti,ab,kw OR (Relapsing Fever):ti,ab,kw OR (Severe Fever with Thrombocytopenia Syndrome):ti,ab,kw OR (Spotted Fever Group Rickettsiosis):ti,ab,kw 446  #10 (Theileriasis):ti,ab,kw OR (Tularemia):ti,ab,kw OR (bacterial vector):ti,ab,kw OR (bacterial vectors):ti,ab,kw OR (Arachnid Vectors):ti,ab,kw 84  #11 (Arachnid Vector):ti,ab,kw OR (Vectors, Arachnid):ti,ab,kw 4  #12 #1 or #2 or #3 or #4 or #5 or #6 or #7 or #8 or #9 or #10 or #11 1065  #13 MeSH descriptor: [Rickettsia] explode all trees 7  #14 (Rickettsia):ti,ab,kw OR (Rickettsia Infections):ti,ab,kw OR (Boutonneuse Fever):ti,ab,kw OR (Rocky Mountain Spotted Fever):ti,ab,kw OR (Typhus, Endemic Flea-Borne):ti,ab,kw 48  #15 (Typhus, Epidemic Louse-Borne):ti,ab,kw OR (Rickettsial Diseases):ti,ab,kw OR (Rickettsial Disease):ti,ab,kw OR (Rickettsiosis):ti,ab,kw OR (Rickettsioses):ti,ab,kw 19  #16 (Rickettsia Infectious):ti,ab,kw OR (Rickettsia Infection):ti,ab,kw OR (Spotted Fever Group Rickettsiosis):ti,ab,kw OR (Spotted Fevers):ti,ab,kw OR (Spotted Fever):ti,ab,kw 40  #17 (spotted fever rickettsiae disease):ti,ab,kw OR (Tick-Borne Lymphadenopathy):ti,ab,kw OR (Tick Borne Lymphadenopathy):ti,ab,kw OR (Tick-Borne Lymphadenopathies):ti,ab,kw OR (Rickettsia slovaca Infections):ti,ab,kw 2  #18 (TIBOLA):ti,ab,kw OR (African Tick-Bite Fever):ti,ab,kw OR (African Tick Bite Fever):ti,ab,kw OR (Flinders Island Spotted Fever):ti,ab,kw OR (Rickettsialpox):ti,ab,kw 0  #19 (Far Eastern Spotted Fever):ti,ab,kw OR (North Asian Tick Typhus):ti,ab,kw OR (Queensland Tick Typhus):ti,ab,kw OR (Boutonneuse Fever):ti,ab,kw OR (Rocky Mountain Spotted Fever):ti,ab,kw 19  #20 (Coxiella burnetiid):ti,ab,kw OR (burnetia burneti):ti,ab,kw OR (coxiella burneti):ti,ab,kw OR (coxiella burnetti):ti,ab,kw OR (coxiella burnettii):ti,ab,kw 3  #21 (coxiella diaporica):ti,ab,kw OR (coxsiella burnetii):ti,ab,kw 0  #22 #13 or #14 or #15 or #16 or #17 or #18 or #19 or #20 or #21 64  #23 MeSH descriptor: [Prevalence] explode all trees 4988  #24 (Prevalences):ti,ab,kw OR (Prevalence):ti,ab,kw OR (Period Prevalences):ti,ab,kw OR (Point Prevalences):ti,ab,kw OR (Epidemiology):ti,ab,kw 92100  #25 (Social Epidemiologies):ti,ab,kw OR (controlled before after studies):ti,ab,kw OR (controlled before and after studies):ti,ab,kw OR (controlled before and after study):ti,ab,kw OR (controlled before-after studies):ti,ab,kw 94136  #26 (effect modifier, epidemiologic):ti,ab,kw OR (effect modifiers (psychology)):ti,ab,kw OR (epidemiologic confounding factors):ti,ab,kw OR (epidemiologic effect modifier):ti,ab,kw OR (epidemiologic factors):ti,ab,kw 1364  #27 (epidemiologic methods):ti,ab,kw OR (epidemiologic research):ti,ab,kw OR (epidemiologic research design):ti,ab,kw OR (epidemiologic studies):ti,ab,kw OR (epidemiologic study characteristics):ti,ab,kw 3717  #28 (epidemiologic study characteristics as topic):ti,ab,kw OR (epidemiologic survey):ti,ab,kw OR (epidemiological research):ti,ab,kw OR (epidemiometry):ti,ab,kw OR (historically controlled study):ti,ab,kw 2721  #29 (interrupted time series analysis):ti,ab,kw OR (precipitating factors):ti,ab,kw OR (sampling studies):ti,ab,kw OR (Epidemiologic Studies):ti,ab,kw OR (Epidemiological Studies):ti,ab,kw 14117  #30 (Epidemiological Study):ti,ab,kw OR (Epidemiologic Study):ti,ab,kw OR (Disease Transmission, Infectious):ti,ab,kw OR (Pathogen Transmission):ti,ab,kw OR (Infectious Disease Transmission):ti,ab,kw 9345  #31 (Communicable Disease Transmission):ti,ab,kw OR (Close-Contact Transmission):ti,ab,kw OR (Close-Contact Transmissions):ti,ab,kw OR (Close-Contact Infectious Disease Transmission):ti,ab,kw OR (Close Contact Infectious Disease Transmission):ti,ab,kw 137  #32 (Horizontal Transmission of Infectious Disease):ti,ab,kw OR (Pathogen Transmission, Horizontal):ti,ab,kw OR (Horizontal Transmission of Infection):ti,ab,kw OR (Person-to-Person Transmission):ti,ab,kw OR (Person to Person Transmission):ti,ab,kw 700  #33 (Droplet Transmission of Infectious Disease):ti,ab,kw OR (Infectious Disease Droplet Transmission):ti,ab,kw OR (Autochthonous Transmission):ti,ab,kw OR (Autochthonous Transmissions):ti,ab,kw OR (Disease Superspreader Event):ti,ab,kw 16  #34 (Disease Superspreader Events):ti,ab,kw OR (Disease Superspreading):ti,ab,kw OR (Disease Superspreadings):ti,ab,kw OR (Community Spread):ti,ab,kw OR (Community Transmission):ti,ab,kw 1636  #35 (Community Transmissions):ti,ab,kw OR (Incidence):ti,ab,kw OR (Incidences):ti,ab,kw OR (Secondary Attack Rate):ti,ab,kw OR (Secondary Attack Rates):ti,ab,kw 140144  #36 (Attack Rate):ti,ab,kw OR (Attack Rates):ti,ab,kw OR (Cumulative Incidences):ti,ab,kw OR (Person-time Rate):ti,ab,kw OR (Person time Rate):ti,ab,kw 7075  #37 (Person-time Rates):ti,ab,kw 34  #38 #23 or #24 or #25 or #26 or #27 or #28 or #29 or #30 or #31 #32 or #33 or #34 or #35 or #36 or #37 310019  #39 MeSH descriptor: [China] explode all trees 5814  #40 (China):ti,ab,kw OR (Sinkiang):ti,ab,kw OR (Inner Mongolia):ti,ab,kw OR (Manchuria):ti,ab,kw OR (Beijing):ti,ab,kw 19121  #41 (Hong Kong):ti,ab,kw OR (Macau):ti,ab,kw OR (Tibet):ti,ab,kw OR (Chinese People's Republic):ti,ab,kw 3316  #42 #39 or #40 or #41 21933  #43 #12 and #22 15  #44 #43 and #38 and #42 1 | 1 |
| Web of Science | 1 Tick (Topic) or Ticks (Topic) or Ixodida (Topic) or Ixodidas (Topic) or Ixodidae (Topic) or Argasidae (Topic) or Argas (Topic) or Ornithodoros (Topic) or Ixodidae (Topic) or Amblyomma (Topic) or Dermacentor (Topic) or Ixodes (Topic) or Rhipicephalus (Topic) or Vector Borne Diseases (Topic) or Ixodoidea (Topic) or Vector Borne Disease (Topic) or Vector-Borne Diseases (Topic) or Vector-Borne Disease (Topic) or Vectorborne Diseases (Topic) or Vectorborne Disease (Topic) or Mosquito-Borne Diseases (Topic) or Mosquito-Borne Disease (Topic) or Mosquito Borne Diseases (Topic) or Mosquito Borne Disease (Topic) or bacterial vector (Topic) or bacterial vectors (Topic) or Tick-Borne Diseases (Topic) or African Swine Fever (Topic) or Anaplasmosis (Topic) or Babesiosis (Topic) or Colorado Tick Fever (Topic) or Ehrlichiosis (Topic) or Encephalitis, Tick-Borne (Topic) or Hemorrhagic Fever, Crimean (Topic) or Hemorrhagic Fever, Omsk (Topic) or Kyasanur Forest Disease (Topic) or Lyme Disease (Topic) or Nairobi Sheep Disease (Topic) or Relapsing Fever (Topic) or Severe Fever with Thrombocytopenia Syndrome (Topic) or Spotted Fever Group Rickettsiosis (Topic) or Theileriasis (Topic) or Tularemia (Topic) or Arachnid Vectors (Topic) or Arachnid Vector (Topic) or Vectors, Arachnid (Topic) 502,093  2 Rickettsia (Topic) or Rickettsia Infections (Topic) or Boutonneuse Fever (Topic) or Rocky Mountain Spotted Fever (Topic) or Typhus, Endemic Flea-Borne (Topic) or Typhus, Epidemic Louse-Borne (Topic) or Rickettsial Diseases (Topic) or Rickettsial Disease (Topic) or Rickettsiosis (Topic) or Rickettsioses (Topic) or Rickettsia Infectious (Topic) or Rickettsia Infection (Topic) or Spotted Fever Group Rickettsiosis (Topic) or Spotted Fever (Topic) or Spotted Fevers (Topic) or spotted fever rickettsiae disease (Topic) or Tick-Borne Lymphadenopathy (Topic) or Tick Borne Lymphadenopathy (Topic) or Tick-Borne Lymphadenopathies (Topic) or Rickettsia slovaca Infections (Topic) or TIBOLA (Topic) or African Tick-Bite Fever (Topic) or African Tick Bite Fever (Topic) or Flinders Island Spotted Fever (Topic) or Rickettsialpox (Topic) or Far Eastern Spotted Fever (Topic) or North Asian Tick Typhus (Topic) or Queensland Tick Typhus (Topic) or Boutonneuse Fever (Topic) or Rocky Mountain Spotted Fever (Topic) or Coxiella burnetiid (Topic) or burnetia burneti (Topic) or coxiella burneti (Topic) or coxiella burnetti (Topic) or coxiella burnettii (Topic) or coxiella diaporica (Topic) or coxsiella burnetii (Topic) 71,903  3 Prevalences (Topic) or Prevalence (Topic) or Period Prevalences (Topic) or Point Prevalences (Topic) or Epidemiology (Topic) or Social Epidemiologies (Topic) or controlled before after studies (Topic) or controlled before and after studies (Topic) or controlled before and after study (Topic) or controlled before-after studies (Topic) or effect modifier, epidemiologic (Topic) or effect modifiers (psychology) (Topic) or epidemiologic confounding factors (Topic) or epidemiologic effect modifier (Topic) or epidemiologic factors (Topic) or epidemiologic methods (Topic) or epidemiologic research (Topic) or epidemiologic research design (Topic) or epidemiologic research design (Topic) or epidemiologic study characteristics (Topic) or epidemiologic study characteristics as topic (Topic) or epidemiologic survey (Topic) or epidemiometry (Topic) or historically controlled study (Topic) or precipitating factors (Topic) or sampling studies (Topic) or Epidemiologic Studies (Topic) or Epidemiologic Studies (Topic) or Epidemiological Study (Topic) or Epidemiologic Study (Topic) or Disease Transmission, Infectious (Topic) or Pathogen Transmission (Topic) or Infectious Disease Transmission (Topic) or Communicable Disease Transmission (Topic) or Close-Contact Transmission (Topic) or Close-Contact Transmissions (Topic) or Close-Contact Infectious Disease Transmission (Topic) or Close Contact Infectious Disease Transmission (Topic) or Horizontal Transmission of Infectious Disease (Topic) or Pathogen Transmission, Horizontal (Topic) or Horizontal Transmission of Infection (Topic) or Person-to-Person Transmission (Topic) or Person to Person Transmission (Topic) or Droplet Transmission of Infectious Disease (Topic) or Infectious Disease Droplet Transmission (Topic) or Autochthonous Transmission (Topic) or Autochthonous Transmissions (Topic) or Disease Superspreader Event (Topic) or Disease Superspreader Events (Topic) or Disease Superspreading (Topic) or Disease Superspreadings (Topic) or Community Transmission (Topic) or Community Transmissions (Topic) or Community Spread (Topic) or Incidence (Topic) or Incidences (Topic) or Secondary Attack Rate (Topic) or Secondary Attack Rates (Topic) or Attack Rate (Topic) or Attack Rates (Topic) or Cumulative Incidences (Topic) or Person-time Rate (Topic) or Person time Rate (Topic) or Person-time Rates (Topic) 8,809,096  4 China (Topic) or Sinkiang (Topic) or Inner Mongolia (Topic) or Manchuria (Topic) or Beijing (Topic) or Hong Kong (Topic) or Macau (Topic) or Tibet (Topic) or Chinese People's Republic (Topic) 1,733,870  5 #31 AND #32 26,395  6 #33 AND #34 AND #35 707 | 707 |
| CNKI | TKA = ('蜱'+'草爬子'+'壁虱') AND TKA = ('立克次体'+'立克次体属'+'立克次体感染'+'立克次体族'+'立克次体科'+'斑疹伤寒'+'地方性蚤传'+'流行性虱传') AND TKA = ('流行病学'+'患病率'+'流行率'+'现患率'+'发病率'+'发生率'+'调查'+'阳性率'+'感染') | 452 |
| VIP | ((((((((任意字段=蜱 OR 任意字段=草爬子) OR 任意字段=壁虱) AND (((((((任意字段=立克次体 OR 任意字段=立克次体属) OR 任意字段=立克次体感染) OR 任意字段=立克次体族) OR 任意字段=立克次体科) OR 任意字段=斑疹伤寒) OR 任意字段=地方性蚤传) OR 任意字段=流行性虱传)))) AND ((((((((任意字段=流行病学 OR 任意字段=患病率) OR 任意字段=流行率) OR 任意字段=现患率) OR 任意字段=发病率) OR 任意字段=发生率) OR 任意字段=调查) OR 任意字段=阳性率) OR 任意字段=感染)))) | 270 |
| CBM | 1)"流行病学"[不加权:扩展] 51574  2) "流行病学"[常用字段:智能] OR "患病率"[常用字段:智能] OR "流行率"[常用字段:智能] OR "现患率"[常用字段:智能] OR "发病率"[常用字段:智能] OR "发生率"[常用字段:智能] OR "调查"[常用字段:智能] OR "阳性率"[常用字段:智能] OR "感染"[常用字段:智能] 9163996 2023-01-09 01:11:06.0  3) ("流行病学"[常用字段:智能] OR "患病率"[常用字段:智能] OR "流行率"[常用字段:智能] OR "现患率"[常用字段:智能] OR "发病率"[常用字段:智能] OR "发生率"[常用字段:智能] OR "调查"[常用字段:智能] OR "阳性率"[常用字段:智能] OR "感染"[常用字段:智能]) OR ("流行病学"[不加权:扩展]) 9163996  4) "立克次体属"[不加权:扩展] 717  5) "立克次体"[常用字段:智能] OR "立克次体属"[常用字段:智能] OR "立克次体感染"[常用字段:智能] OR "立克次体族"[常用字段:智能] OR "立克次体科"[常用字段:智能] OR "斑疹伤寒"[常用字段:智能] OR "地方性蚤传"[常用字段:智能] OR "流行性虱传"[常用字段:智能] 21633  6) ("立克次体"[常用字段:智能] OR "立克次体属"[常用字段:智能] OR "立克次体感染"[常用字段:智能] OR "立克次体族"[常用字段:智能] OR "立克次体科"[常用字段:智能] OR "斑疹伤寒"[常用字段:智能] OR "地方性蚤传"[常用字段:智能] OR "流行性虱传"[常用字段:智能]) OR ("立克次体属"[不加权:扩展]) 21633  7) "蜱"[不加权:扩展] 1813  8)"蜱"[常用字段:智能] OR "草爬子"[常用字段:智能] OR "壁虱"[常用字段:智能] 48638  9) ("蜱"[常用字段:智能] OR "草爬子"[常用字段:智能] OR "壁虱"[常用字段:智能]) OR ("蜱"[不加权:扩展]) 48638  10) (("蜱"[常用字段:智能] OR "草爬子"[常用字段:智能] OR "壁虱"[常用字段:智能]) OR ("蜱"[不加权:扩展])) AND (("立克次体"[常用字段:智能] OR "立克次体属"[常用字段:智能] OR "立克次体感染"[常用字段:智能] OR "立克次体族"[常用字段:智能] OR "立克次体科"[常用字段:智能] OR "斑疹伤寒"[常用字段:智能] OR "地方性蚤传"[常用字段:智能] OR "流行性虱传"[常用字段:智能]) OR ("立克次体属"[不加权:扩展])) AND (("流行病学"[常用字段:智能] OR "患病率"[常用字段:智能] OR "流行率"[常用字段:智能] OR "现患率"[常用字段:智能] OR "发病率"[常用字段:智能] OR "发生率"[常用字段:智能] OR "调查"[常用字段:智能] OR "阳性率"[常用字段:智能] OR "感染"[常用字段:智能]) OR ("流行病学"[不加权:扩展])) 3664 | 309 |
| WanFang | 主题:(蜱 or 草爬子 or 壁虱) and 主题:(立克次体 or 立克次体属 or 立克次体感染 or 立克次体族 or 立克次体科 or 斑疹伤寒 or地方性蚤传 or 流行性虱传) and 主题:(流行病学 or 患病率 or 流行率 or 现患率 or 发病率 or 发生率 or 调查 or 阳性率 or 感染) | 1062 |
