# Supplementary material for: A systematic review and meta-analysis of the prevalence of tick-borne SFGR in China from 2000 to 2022
Source: PLoS Negl Trop Dis. 2024 Oct 9;18(10):e0012550. doi: 10.1371/journal.pntd.0012550 (PMC11463837; doi:10.1371/journal.pntd.0012550)
Supplement: S2 Table — (DOCX) [file pntd.0012550.s002.docx]

**Table 2. Studies included in systematic literature review contributing to tick-borne SFGR positive rate analyses**

| Study (n) | | Year(s) of  data  collection | Study  Denominator | Quality evaluation score | Positive rate (%) | Source of ticks | Numbers of tick species | Tick species | Numbers of SFGR | SFGR category |
| --- | --- | --- | --- | --- | --- | --- | --- | --- | --- | --- |
| **Northern area(43)** | **-** | **Total** |  |  |  |  |  |  |  |  |
| Shengyue 2020 [1] | 2016 | 379 | 3 | 63.90 | Free Parasitic | 3 | Dermacentor nuttalli, Dermacentor silvarum, Ixodes persulcatus | 3 | Rickettsia raoultii, Candidatus Rickettsia tarasevichiae, Rickettsia Sibirica |  |
| Wangzhuo 2018 [2] | 2014 | 1224 | 3 | 12.44 | Free  Parasitic | 5 | Haemaphysalis concinna, Haemaphysalis japonica, Haemaphysalis Iongicornis, Dermacentor silvarum, Ixodes persulcatus | 3 | Rickettsia heilongjiangiensis, Rickettsia raoultii, Candidatus Rickettsia hebeiii |  |
| Xujun 2016 [3] | 2014 | 392 | 3 | 36.84 | Free | 3 | Rhipicephalus sanguineus, Hyalomma asiaticum, Dermaentor marginatus | 1 | Rickettsia raoultii |  |
| Luodan 2016 [4] | 2015 | 253 | 2 | 16.21 | Free  Parasitic | 4 | Rhipicephalus sanguineus, Hyalomma asiaticum, Dermaentor marginatus, Haemaphysalis erinacei turanica | 1 | Rickettsia burneti |  |
| Zhoulei 2017 [5] | 2014 | 320 | 2 | 47.50 | Free | 3 | Ixodes persulcatus, Dermacentor silvarum, Haemaphysalis concinna | 3 | Candidatus Rickettsia tarasevichiae, Rickettsia raoultii, Rickettsia heilongjiangiensis |  |
| Wulanyatu 2018 [6] | 2017 | 2000 | 2 | 59.00 | Parasitic | 5 | Dermacentor nuttalli, Rhipicephalus pumilio, Rhipicephalus sanguineus, Haemaphysalis bispinosa, Haemaphysalis Iongicornis | 0 |  |  |
| Guizheng 2021 [7] | 2019 | 264 | 2 | 82.57 | Parasitic | 1 | Dermacentor nuttalli | 5 | Rickettsia raoultii, Rickettsia parkeri, Rickettsia Sibirica, Rickettsia massiliae, Rickettsia rhipicephali |  |
| Tangfang 2017 [8] | 2017 | 723 | 2 | 39.14 | FreeParasitic | 1 | Ixodes persulcatus | 3 | Candidatus Rickettsia tarasevichiae, Rickettsia raoultii, Rickettsia heilongjiangiensis |  |
| Haoyongjian 2003 [9] | 2003 | 683 | 2 | 30.61 | FreeParasitic | 3 | Dermacentor silvarum, Haemaphysalis concinna, Ixodes persulcatus | 1 | Rickettsia Sibirica |  |
| Huyang 2019 [10] | 2019 | 133 | 2 | 64.70 | FreeParasitic | 3 | Haemaphysalis, Dermacentor, Ixodes persulcatus | 0 |  |  |
| Zouyaxue 2011 [11] | 2011 | 315 | 2 | 7.94 | FreeParasitic | 1 | Haemaphysalis Iongicornis | 3 | Rickettsia sp.Japanese strain, Rickettsia sp.Fujian strain, Rickettsia heilongjiangiensis |  |
| Xiaochendong 2021 [12] | 2020 | 585 | 2 | 39.70 | Free | 5 | Dermacentor silvarum, Haemaphysalis concinna, Haemaphysalis japonica, Haemaphysalis Iongicornis, Ixodes persulcatus | 5 | Candidatus Rickettsia longicornii, Rickettsia raoultii, Rickettsia heilongjiangiensis, Candidatus Rickettsia tarasevichiae, Rickettsia monacensi |  |
| Liuchengcheng 2020 [13] | 2018 | 818 | 2 | 7.80 | Free | 3 | Haemaphysalis concinna, Ixodes persulcatus, Dermacentor silvarum | 2 | Rickettsia raoultii, Candidatus Rickettsia tarasevichiae |  |
| Lijixu 2019 [14] | 2017 | 3079 | 2 | 17.70 | FreeParasitic | 1 | Haemaphysalis Iongicornis | 1 | Candidatus Rickettsia taraseviehia |  |
| Gaoyang 2022 [15] | 2019 | 919 | 2 | 57.23 | FreeParasitic | 1 | Dermacentor nuttalli | 1 | Rickettsia raoultii |  |
| Zhanglijuan 2006 [16] | 1999 | 20 | 2 | 63.33 | Parasitic | 4 | Rhipicephalus turanicus, Dermaentor marginatus, Dermacentor niveus, Hyalomma asiaticum | 4 | Candidatus Rickettsia barbariae, Rickettsia raoultii, Rickettsia conorii, Rickettsia massiliae |  |
| Fanyadi 2016 [17] | 2013 | 215 | 2 | 23.15 | FreeParasitic | 2 | Dermacentor silvarum, Dermaentor marginatus | 1 | Rickettsia raoultii |  |
| Zhanglijuan 2006 [18] | 1999 | 230 | 3 | 12.35 | Parasitic | 2 | Rhipicephalus turanicus, Rhipicephalus sanguineus | 2 | Candidatus Rickettsia barbariae, uncultured Rickettsia sp.(KT284716.1, KT284715.1) |  |
| Sunsurong 2020 [19] | 2017 | 173 | 2 | 51.50 | FreeParasitic | 7 | Dermacentor silvarum, Dermacentor nuttalli, Dermacentor pavlovskyi, Hyalomma anatolicum anatolicum, Hyalomma rufipes, Hyalomma asiaticum, Rhipicephalus sanguineus | 6 | Rickettsia raoultii, Rickettsia Sibirica, Rickettsia slovaca, Rickettsia aeschlimannii, uncultured Candidatus Rickettsia, Candidatus Rickettsia barbariae |  |
| Xiyu 2020 [20] | 2019 | 1340 | 3 | 1.00 | Free | 1 | Hyalomma asiaticum | 1 | Rickettsia raoultii |  |
| Sunxiang 2013 [21] | 2012 | 311 | 2 | 30.61 | FreeParasitic | 3 | Dermacentor silvarum, Haemaphysalis concinna, Ixodes persulcatus | 1 | Rickettsia montaneisi |  |
| Sunxiang 2017 [22] | 2015 | 510 | 2 | 7.42 | FreeParasitic | 1 | Haemaphysalis Iongicornis | 4 | Candidatus Rickettsia hebeiii, Rickettsia sp. FUJ98, Rickettsia heilongjiangiensis, Rickettsia japonica |  |
| Quzhiqiang 2019 [23] | 2018 | 2079 | 2 | 44.44 | Parasitic | 1 | Ixodes persulcatus | 0 |  |  |
| Lijing 2017 [24] | 2015 | 100 | 2 | 3.08 | FreeParasitic | 3 | Haemaphysalis qinghaiensis, Dermacentor nuttalli, Dermacentor silvarum | 2 | Rickettsia Sibirica, Rickettsia heilongjiangiensis |  |
| Haoyongjian 2005 [25] | 2005 | 683 | 2 | 1.90 | FreeParasitic | 1 | Haemaphysalis Iongicornis | 4 | Candidatus Rickettsia hebeiii, Rickettsia sp.(KC888951.1), Rickettsia sp.hl-93, Rickettsia japonica |  |
| Hanqian 2019 [26] | 2016 | 395 | 2 | 22.25 | FreeParasitic | 2 | Ixodes persulcatus, Haemaphysalis concinna | 1 | Rickettsia japonica |  |
| Fengshuai 2013 [27] | 2012 | 1227 | 2 | 51.51 | Free | 4 | Dermacentor silvarum, Haemaphysalis concinna, Ixodes persulcatus, Haemaphysalis japonica | 1 | Rickettsia heilongjiangiensis |  |
| Jiangliping 2010 [28] | 2007 | 232 | 3 | 38.18 | Parasitic | 1 | Haemaphysalis Iongicornis | 1 | Candidatus R.longicornii |  |
| Libingbing 2018 [29] | 2017 | 83 | 2 | 3.90 | Free | 3 | Dermacentor nuttalli, Haemaphysalis qinghaiensis, Dermacentor silvarum | 4 | Rickettsia slovaca, Rickettsia raoultii, Rickettsia Sibirica, Rickettsia sp.DnS28 |  |
| Denghua 2014 [30] | 2013 | 18 | 2 | 9.80 | FreeParasitic | 1 | Haemaphysalis qinghaiensis | 3 | Rickettsia sp. FUJ98, Rickettsia heilongjiangiensis HL-93,HL-054, Rickettsia japonica |  |
| Gudengan 2018 [31] | 2017 | 148 | 2 | 64.48 | Free | 3 | Dermacentor silvarum, Haemaphysalis concinna, Ixodes persulcatus | 1 | Rickettsia raoultii |  |
| Liying 2014 [32] | 2011 | 10304 | 3 | 15.76 | Free | 4 | Ixodes persulcatus, Dermacentor silvarum, Haemaphysalis concinna, Haemaphysalis japonica | 3 | Rickettsia raoultii, Candidatus Rickettsia rara, Candidatus Rickettsia tarasevichiae |  |
| Wangzhuo 2018 [33] | 2013 | 368 | 2 | 10.29 | FreeParasitic | 4 | Ixodes persulcatus,Dermacentor silvarum, Haemaphysalis concinna, Dermacentor japonica | 3 | Rickettsia sibirica, Rickettsia heilongjiangensis, Rickettsia hulinii |  |
| Fuweiming 2021 [34] | 2017 | 418 | 2 | 16.81 | Free | 4 | Ixodes persulcatus,Dermacentor silvarum, Haemaphysalis concinna, Haemaphysalis japonica | 0 |  |  |
| Wuyimin 2003 [35] | 2003 | 6619 | 3 | 1.59 | Free | 2 | Dermacentor silvarum, Ixodes persulcatus | 1 | Rickettsia heilongjiangiensis |  |
| Lijixu 2021 [36] | 2019 | 55 | 2 | 6.12 | FreeParasitic | 5 | Dermacentor nuttalli, Dermacentor silvarum, Haemaphysalis concinna, Haemaphysalis Iongicornis, Ixodes persulcatus | 3 | Candidatus Rickettsia vini, Candidatus Rickettsia jingxinensis, Rickettsia raoultii |  |
| Gaoyue 2019 [37] | 2016 | 1294 | 2 | 28.80 | FreeParasitic | 5 | Dermacentor nuttalli, Dermacentor silvarum, Haemaphysalis concinna, Haemaphysalis Iongicornis, Ixodes persulcatus | 2 | Candidatus Rickettsia tarasevichiae, Rickettsia raoultii |  |
| Yangjufeng 2019 [38] | 2014 | 1548 | 2 | 11.00 | FreeParasitic | 1 | Dermacentor silvarum | 2 | Rickettsia raoultii, Rickettsia slovaca |  |
| Yenannan 2018 [39] | 2014 | 1343 | 2 | 23.33 | Parasitic | 1 | Dermacentor silvarum | 2 | Rickettsia sp.JL-02, Rickettsia raoultii |  |
| Fuyingqun 2015 [40] | 2013 | 165 | 2 | 11.80 | Parasitic | 1 | Rhipicephalus sanguineus | 3 | Candidatus Rickettsia barbariae, Rickettsia massiliae, Rickettsia conorii |  |
| Sunxiufeng 2007 [41] | 2007 | 3926 | 3 | 44.50 | FreeParasitic | 5 | Haemaphysalis qinghaiensis, Dermacentor abaensis, Dermacentor silvarum, Dermacentor nuttalli, Ixodes crenulatus | 5 | Rickettsia sibirica subspecies sibirica, Rickettsia raoultii, Candidatus Rickettsia tibetani, Candidatus Rickettsia gannanii Y27,F107, uncultured Rickettsia sp.10CYF |  |
| Chenxia 2022 [42] | 2021 | 351 | 2 | 18.50 | Free | 1 | Haemaphysalis qinghaiensis | 1 | uncultured Rickettsia |  |
| Shiqi 2020 [43] | 2019 | 63 | 2 | 54.14 | FreeParasitic | 1 | Haemaphysalis qinghaiensis | 1 | Rickettsia raoultii |  |
| **Southern area (14)** | **-** | **Total** |  |  |  |  |  |  |  |  |
| Liuhuanhuan, 2016 [44] | 2015 | 2928 | 2 | 63.93 | FreeParasitic | 4 | Rhipicephalus microplus, Ixodidae ovatus, Ixodes acutitarsus Karsch, Dermacentor everestianus | 5 | Rickettsia raoultii, Candidatus Rickettsia longicornii, uncultured Rickettsia sp.9 (MF134885), uncultured Rickettsia sp.CNH17-7 (MK236551), uncultured Rickettsia sp.5-15 (MN631235) |  |
| Wangqian 2021 [45] | 2019 | 305 | 2 | 49.80 | FreeParasitic | 2 | Haemaphysalis qinghaiensis, Dermacentor everestianus | 1 | uncultured Rickettsia, Rickettsia raoultii |  |
| Sunjimin 2015 [46] | 2011 | 292 | 2 | 88.00 | Parasitic | 3 | Boophilus microplus, Haemaphysalis cornigera, Ixodes granulatus | 4 | Rickettsia honei, Rickettsia africa, Rickettsia slovaca, Rickettsia Sibirica |  |
| Shaojianwei 2020 [47] | 2020 | 1286 | 2 | 93.30 | FreeParasitic | 1 | Haemaphysalis Iongicornis | 2 | Rickettsia heilongjiangiensis, Rickettsia massiliae |  |
| Chengtianzhan 2012 [48] | 2012 | 200 | 2 | 4.30 | FreeParasitic | 4 | Haemaphysalis Iongicornis, Haemaphysalis cornigera, Rhipicephalus microplus, Haemaphysalis concinna | 4 | Rickettsia japonica, Rickettsia rhipicephali, Rickettsia massiliae, Rickettsia raoultii |  |
| Linbaoshan 2022 [49] | 2018 | 818 | 2 | 5.46 | Parasitic | 3 | Haemaphysalis Iongicornis, Rhipicephalus haemaphysaloides, Rhipicephalus sanguineus | 2 | Rickettsia sp.71-8, Rickettsia sp.Fujian strain |  |
| Chien-Ming Shih 2021 [50] | 2021 | 247 | 2 | 3.61 | FreeParasitic | 1 | Haemaphysalis Iongicornis | 3 | Rickettsia japonica, Rickettsia hulinensis, Rickettsia heilongjiangensis |  |
| Fengli 2014 [51] | 2014 | 60 | 2 | 2.80 | FreeParasitic | 4 | Haemaphysalis sinensis, Haemaphysalis Iongicornis, Rhipicephalus sanguineus, Boophilus microplus | 1 | Rickettsia heilongjiangii |  |
| Wurelihazi  hazihan 2019 [52] | 2017 | 178 | 2 | 36.10 | FreeParasitic | 9 | Rhipicephalus microplus, Haemaphysalis Iongicornis, Amblyomma javanense, Amblyomma testudinarium, Ixodes granulatus, Ixodidae ovatus, Amblyomma geoemydae, Haemaphysalis hystricis, Rhipicephalus haemaphysaloides | 2 | Candidatus Rickettsia jingxinensis, a potential novel species Rickettsia sp.sw |  |
| Yuantingting 2021 [53] | 2018 | 581 | 2 | 7.53 | Parasitic | 10 | Haemaphysalis Iongicornis, Rhipicephalus haemaphysaloides, Amblyomma testudinarium, Ixodes sinensis, Rhipicephalus microplus, Ixodes granulatus, Haemaphysalis yeni, Dermacentor taiwanensis, Haemaphysalis hystricis, Hyalomma asiaticum | 0 |  |  |
| Zhangxing 2018 [54] | 2014 | 188 | 2 | 9.90 | Parasitic | 2 | Dermacentor everestianus, Haemaphysalis qinghaiensis | 4 | Rickettsia raoultii, Candidatus Rickettsia longicornii, uncultured Rickettsia |  |
| Hanrong 2017 [55] | 2016 | 860 | 2 | 4.86 | Parasitic | 1 | Ixodes granulatus | 2 | Rickettsia parkeri-like, Rickettsia felis |  |
| Yangjifei 2016 [56] | 2011 | 1583 | 3 | 12.60 | Parasitic | 7 | Haemaphysalis formosensis, Haemaphysalis hystricis, Dermacentor steini, Rhipicephalus sanguineus, Rhipicephalus microplus, Haemaphysalis montgomeryi, Ixodidae ovatus | 2 | Rickettsia tamurae strain AT-197.3, Candidatus Rickettsia longicornii isolate ROK-HL727 |  |
| Heyongcai 2021 [57] | 2022 | 266 | 2 | 33.50 | FreeParasitic | 4 | Haemaphysalis Iongicornis, Haemaphysalis flavus, Haemaphysalis doenitzi, Haemaphysalis hystricis | 2 | uncultured Rickettsia sp.clone Y27-1(KT921891), uncultured Rickettsia (KT921894) |  |

**References**

1. Sheng Y, Deng H, Gao Y, Li Y, Zhu J, Niu T, et al. A survey of tick-carried spotted fever group Rickettsia in Inner Mongolia from 2019 to 2020. Chinese Journal of Frontier Health and Quarantine. 2021;44(03): 168-170.

2. Wang Z, Wang JF, Yu M, Xing YP, Feng L, Yang YJ, et al. Molecular epidemiological studies on spotted fever group rickettsia in ticks from Northeastern China. Chinese Journal of Vector Biology and Contro. 2018;29(4): 344-347.

3. Xu J, Wang AD, Luo D, Xu XL, Dai L, Yang J, et al. Molecular epidemiological study of the spotted fever group Rickettsia in free-living ticks in the wetlands of Aibi Lake, China-Kazakhstan border. Chinese Journal of Veterinary Medicine. 2016;52(8): 18-20.

4. Luo D, Yin XP, Wang AD, Tian YH, Liang Z, Ba T, et al. The first detection of Coxiella burnetii DNA from Hyalomma asiaticum at Alataw pass, China-Kazakhstan border area. Disease Surveillance. 2016;31(10): 814-816.

5. Zhou L, Tang F, Luan J, Liu W. Investigation on Rickettsia spotted fever carried by ticks in Qigan area, Inner Mongolia. Chinese Journal of Frontier Health and Quarantine. 2017;40(02): 96-99.

6. Wulantuya, Gao W, Guo XH, Guo SC, Aribenjirigala. Study on the correlation between tick species and Rickettsiella in western Inner Mongolia. Modern Preventive Medicine. 2018;45(11): 2059-2062+2072.

7. Gui Z, Yu JF, Mu L. DNA detection and genotype distribution of spotted fever group Rickettsiae carried by dermacentor on grassland in some areas of Inner Mongolia. Journal of Jilin University(Medicine Edition). 2021;47(01): 210-215.

8. Tang F, Jiang LF, Liu L, Yang J, Luan J, Guo W, et al. Investigation of spotted fever group Rickettsia carried by ixodes in the Greater Hinggan Mountains of Inner Mongolia. International Journal of Epidemiology and Infectious Disease. 2017;44(06): 427-429.

9. Hao YJ, Cao WC, Gao SP, Zhang PH, Zhao QM, Yang H, et al. Investigation on the natural foci of Rickettsia febris in Changbai Mountain area of Jilin Province. Chinese Journal of Epidemiology. 2003(12): 62-64.

10. Hu Y, Qi K, Cao H, Cong YZ. Investigation on rickettsia infection of three ticks in Jiangshan Scenic area of Harbin. Veterinary Orientation. 2019(21): 80.

11. Zou YX, Jia QH, Liu PP, Liu Q, Gao HW, CHen LF. Molecular epidemiological of spotted fever group Rickettsia from ticks in Tangshan area. Chinese Journal of Veterinary Science. 2011;31(12): 1729-1732.

12. Xiao CD, Yuan DB, Yin NC, Zhang LY, CHen L, Hao LL. Molecular Detection of SFGR in Ticks Collected from Yaks in Jiulong County of Sichuan Province,China. Chinese Journal of Veterinary Parasitology. 2023;31(5): 159-167.

13. Liu CC, Tang TC, Ta Y, Lin BS, Yuan DB, Guo L, et al. Molecular detection of spotted fever group rickettsiae-infected ticks collected from yaks in Shiqu County of Sichuan Province, China. Chinese Journal of Zoonoses. 2020;36(1): 50-55.

14. Li JX, Roh J, Park W, Pu W, Jin GJ, Wu ZG, et al. Investigation and research on ticks carrying spotted fever group rickettsia in the border area of Tumen River Basin. Chinese Journal of Preventive Medicine. 2019;53(11): 1130-1135.

15. Gao YF, Xu SQ, Wang LN, Guo WP, Liu M. Detection and phylogenetic analysis of spotted fever group rickettsiae in ticks in Saihanba National Nature Reserve,China. Chinese Journal of Vector Biology and Control. 2022;33(2): 252-257.

16. Zhang LJ, Zhang JS, Fu XP, Luan MC. The new tick-borne spotted fever group rickettsia exists in ticks from Lianping county of Guangdong province. Chinese Journal of Zoonoses. 2006;22(8): 697-700.

17. Fan YD, Hu JG, Cui XM, Guo CT, Wo Y, Zhang XA, et al. CO-INFECTION OF SPOTTED FEVER GROUP RICKETTSIAE AND SEVERE FEVER WITH THROMBOCYTOPENIA SYNDROME VIRUS IN TICKS IN EASTERN CENTRAL CHINA. Acta Parasitology et Medica Entomologica Sinica. 2016;23(2): 86-90.

18. Zhang LJ, Zhang JS, Fu XP, Luan MC. First identification of a rickettsia closely related to R. Heilongjiangii and R. Massilliae in South China. Infectious Disease Information. 2006;19(2): 65-67.

19. Sun SR, Wang SY, Shi S, Zhang YJ, Zhang JY, Yuan YL, et al. Investigation and genetic evolution of Rickettsial infection in dermacentor grassland on the southern slope of the Wusu Tianshan Mountains in Xinjiang. Journal of Xinjiang University(Natural Science Edition in Chinese and English). 2020;37(02): 190-196.

20. Xi Y, Du YC, Han S, Chen Z, WU JY. Identification of tick species and epidemiology of spotted fever group Rickettsia in parts of Southern Xinjiang. Chinese Journal of Veterinary Science. 2020;40(4): 740-747.

21. Sun X, Zhang GL, Liu R, Liu XM, Zhao Y, Zheng Z. Molecular epidemiology of Rickettsia raoultii in Xinjiang. Chinese Journal of Epidemiology. 2013;34(07): 756-757.

22. Sun X, Zhang GL, Zheng Z, Liu R, Qiu EC. Detection and sequence analysis of Candidatus Rickettsia barbariae from fanhead ticks in Xinjiang. Journal of Parasites and Medical Entomology. 2017;24(03): 141-147.

23. Qu GQ, Lin HL, XU XF, Malike A, Luo Y, Ma Z, et al. Molecular epidemiological study on the spotted fever rickettsia in Xinjiang. Journal of Gansu Agricultural University. 2019;54(5): 10-16.

24. Li J, Yang Y, Yin XP, Liu LJ, Wang JC, Zhang XL, et al. Molecular epidemiological characteristics of tick-borne Rickettsiella at Alashankou port in Xinjiang. Chinese Journal of Frontier Health and Quarantine. 2017;40(03): 170-172.

25. Hao YJ, Cao WC, Gao SP, Zhang PH, Zhao QM, Yang H, et al. The investigation of natural focus of tick-borne spotted fever in a military base. Chinese Journal of Disease Control & Prevention. 2005;9(3): 243-245.

26. Han Q, Xu HB, Feng Y, Tian JH, Zhang SZ, Wu M, et al. Investigation on Rickettsia and Ehrlichia carried by ticks in Ganzhou City, Jiangxi Province. Chinese Journal of zoonoses. 2019;35(06): 518-524.

27. Feng S, Wu H, Zhang LW, Lu CX, Zhang CL, Li ZP, et al. Molecular epidemiology of Rickettsial group tick-borne spotted fever in western mountainous area of Hebei Province. Chinese Journal of vector Biology and Control. 2013;24(04): 308-312.

28. Jiang LP, Meng Z, Cui QR, Dong WS, Ling F, Wang Z. Detection of rOmpA and gltA genes of spotted fever group rickettsiae from tick specimens in Zhejiang province. Chinese Journal of Vector Biology and Control. 2010;21(4): 350-352.

29. Li BB, Yang PF, Liu CC, Li SS, Liu L, Jin J, et al. Detection and phylogenetic analysis of rickettsiae from tick in Huai'an. Modern Preventive Medicine. 2018;45(14): 2641-2646.

30. Deng H, Gao YF, Cheng XL, Gao YF. Detection and sequence analysis on nucleic acid of Rickettsia japonica in ticks of Manzhouli port. Chinese Journal of Frontier Health and Quarantine. 2014;37(5): 336-338.

31. Gu DA, Zhang M, Chen HN, Chen XL, Ma ZH, Gui GP. Survey of tick distribution and tick-borne pathogens in hilly scenic spots of Suzhou, China. Shanghai Journal of Preventive Medicine. 2018;30(8): 652-655.

32. Li Y, Li ZK, Chen G, Kang M, Liu DX, Zhang YM. Identification and phylogenetic analysis of spotted fever group Rickettsia ssolated from Qinghai province. Chinese Journal of Veterinary Science. 2014;34(12): 1956-1961.

33. Wang Z, Wang JW, Yang SJ, Yu M, Kou QH, Feng L, et al. Molecular detection of spotted fever group rickettsiae in Haemaphysalis longicornis from the eastern mountains of Liaoning Province, China. Journal of Parasitic Biology. 2018;13(6): 609-611.

34. Fu WM, Jiao D, Ding SL, Ju WD, Xu N, Geng C, et al. Investigation of Spotted Fever Group Rickettsia in Ticks in the Russian National village of Xunke Coun-ty,Heilongjiang Provice. Port Health Control. 2021;26(4): 60-63.

35. Wu YM, Liu GP, Wei AM, HU LM, Zhang ZQ, Cai ZL, et al. Investigation of natural foci of tick-borne spotted fever in some areas. Chinese Journal of Public Health. 2003;19(9): 1043-1044.

36. Li JX, Piao W, Jin GJ. Transovarial transmission of a new genotype Candidatus Rickettsia longicornii of spotted fever group Rickettsia in Haemaphysalis longicornis. Chinese Journal of Vector Biology and Control. 2021;32(2): 139-143.

37. Gao Y, Li S, Wang LF, Wei F. Epidemiological investigation of tick-borne Rickettsial in Qinghai Province. Heilongjiang Animal Husbandry and Veterinary Medicine. 2019(18): 65-69.

38. Yang JF, Liu QH, Du YC, Li XL, Tian HL, Guo RP, et al. Phylogenetic Analysis and Infection Rate on Tick-borne Spotted Fever Group Rickettsia in Haemaphysalis qinghaiensis. Chinese Journal of Veterinary Medicine. 2019;55(12): 19-21,26.

39. Ye NN, Fei XC, S.H. S. Investigation of the ticks in border trade regions of Heihe. Chinese Journal of Zoonoses. 2018;34(8): 761-767.

40. Fu YQ, Lu TT, Hou D, Liang HJ, Yang J, Yang Y, et al. Investigation of ticks and carried pathogens on Heixiazi island. Chinese Journal of Frontier Health and Quarantine. 2015;38(2): 119-123.

41. Sun XF, Ding SL, Hu MX, Guo XM, ZHao G. Study on Tick-borne Infection at Heilongjiang Port. Chinese Journal of Frontier Health and Quarantine. 2007;30(3): 154-156.

42. Chen X, Niu L, Liang HJ, Fei LJ, Luan P, Sun TH. Surveillance of ticks and tick-borne pathogens at Fuyuan port in Heilongjiang province from 2019 to 2021. Chinese Journal of Frontier Health and Quarantine. 2022;45(5): 357-360.

43. Shi Q, Zhang Q, Liang HJ, Zhang XL, Yang Y, Liu YY, et al. Molecular epidemiological characterization of tick-borne Rickettsia at Luobei port, Heilongjiang province. Chinese Journal of Frontier Health and Quarantine. 2020;43(4): 262-263.

44. Liu H, Li Q, Zhang X, Li Z, Wang Z, Song M, et al. Characterization of rickettsiae in ticks in northeastern China. Parasit Vectors. 2016;9(1): 498.

45. Wang Q, Guo WB, Pan YS, Jiang BG, Du CH, Que TC, et al. Detection of Novel Spotted Fever Group Rickettsiae (Rickettsiales: Rickettsiaceae) in Ticks (Acari: Ixodidae) in Southwestern China. J Med Entomol. 2021;58(3): 1363-1369.

46. Sun J, Lin J, Gong Z, Chang Y, Ye X, Gu S, et al. Detection of spotted fever group Rickettsiae in ticks from Zhejiang Province, China. Exp Appl Acarol. 2015;65(3): 403-411.

47. Shao JW, Zhang XL, Li WJ, Huang HL, Yan J. Distribution and molecular characterization of rickettsiae in ticks in Harbin area of Northeastern China. PLoS Negl Trop Dis. 2020;14(6): e0008342.

48. Tian ZC, Liu GY, Shen H, Xie JR, Luo J, Tian MY. First report on the occurrence of Rickettsia slovaca and Rickettsia raoultii in Dermacentor silvarum in China. Parasit Vectors. 2012;5: 19.

49. Lin B, Ta Y, Hao L. High prevalence of spotted fever group rickettsiae in ticks collected from yaks (Bos grunniens) in Shiqu county, eastern Tibetan Plateau, China. Front Microbiol. 2022;13: 968793.

50. Shin CM, Yang PW, Chao LL. Molecular Detection and Genetic Identification of Rickettsia Infection in Ixodes granulatus Ticks, an Incriminated Vector for Geographical Transmission in Taiwan. Microorganisms. 2021;9(6):

51. Feng L, Wang Z, Yang J, Yu M, Wang LQ, QIu GB, et al. Molecular detection of spotted fever group rickettsia in Dermacentor silvarum from the Xunke Area,China. Chinese Journal of Zoonoses. 2014(10): 1020-1023.

52. Hazihan W, Dong Z, Guo L, Rizabek K, Askar D, Gulzhan K, et al. Molecular detection of spotted fever group rickettsiae in ticks parasitizing pet dogs in Shihezi City, northwestern China. Exp Appl Acarol. 2019;77(1): 73-81.

53. Yuan TT, Du CH, Xia LY, Que TC, von Fricken ME, Jiang BG, et al. Molecular evidence of Candidatus Rickettsia longicornii and a novel Rickettsia strain from ticks in Southern China. Ticks Tick Borne Dis. 2021;12(3): 101679.

54. Zhang X, Geng J, Du J, Wang Y, Qian W, Zheng A, et al. Molecular Identification of Rickettsia Species in Haemaphysalis Ticks Collected from Southwest China. Vector Borne Zoonotic Dis. 2018;18(12): 663-668.

55. Han R, Yang J, Niu Q, Liu Z, Chen Z, Kan W, et al. Molecular prevalence of spotted fever group rickettsiae in ticks from Qinghai Province, northwestern China. Infect Genet Evol. 2018;57: 1-7.

56. Yang J, Tian Z, Liu Z, Niu Q, Han R, Li Y, et al. Novel spotted fever group rickettsiae in Haemaphysalis qinghaiensis ticks from Gansu, Northwest China. Parasit Vectors. 2016;9: 146.

57. Díaz-Sánchez AA, Chilton NB, Roblejo-Arias L, Fonseca-Rodríguez O, Marrero-Perera R, Diyes CP, et al. Molecular detection and identification of spotted fever group rickettsiae in ticks collected from horses in Cuba. Med Vet Entomol. 2021;35(2): 207-212.
